# Molecular understandings on the activation of light hydrocarbons over heterogeneous catalysts

**DOI:** 10.1039/c5sc01227a

**Published:** 2015-06-12

**Authors:** Zhi-Jian Zhao, Cheng-chau Chiu, Jinlong Gong

**Affiliations:** a Key Laboratory for Green Chemical Technology of Ministry of Education , School of Chemical Engineering and Technology , Tianjin University , Collaborative Innovation Center of Chemical Science and Engineering , Tianjin 300072 , China . Email: jlgong@tju.edu.cn ; Fax: +86-22-87401818; b Department Chemie , Technische Universität München , 85747 Garching , Germany

## Abstract

Due to the depletion of petroleum and the recent shale gas revolution, the dropping of the price for light alkanes makes alkanes an attractive feedstock for the production of light alkenes and other valuable chemicals. Understanding the mechanism for the activation of C–H bonds in hydrocarbons provides fundamental insights into this process and a guideline for the optimization of catalysts used for the processing of light alkanes. In the last two decades, density functional theory (DFT) has become a powerful tool to explore elementary steps and mechanisms of many heterogeneously catalyzed processes at the atomic scale. This review describes recent progress on computational understanding of heterogeneous catalytic dehydrogenation reactions of light alkanes. We start with a short description on basic concepts and principles of DFT as well as its application in heterogeneous catalysis. The activation of C–H bonds over transition metal and alloy surfaces are then discussed in detail, followed by C–H activation over oxides, zeolites and catalysts with single atoms as active sites. The origins of coking formation are also discussed followed by a perspective on directions of future research.

## Abstract 2

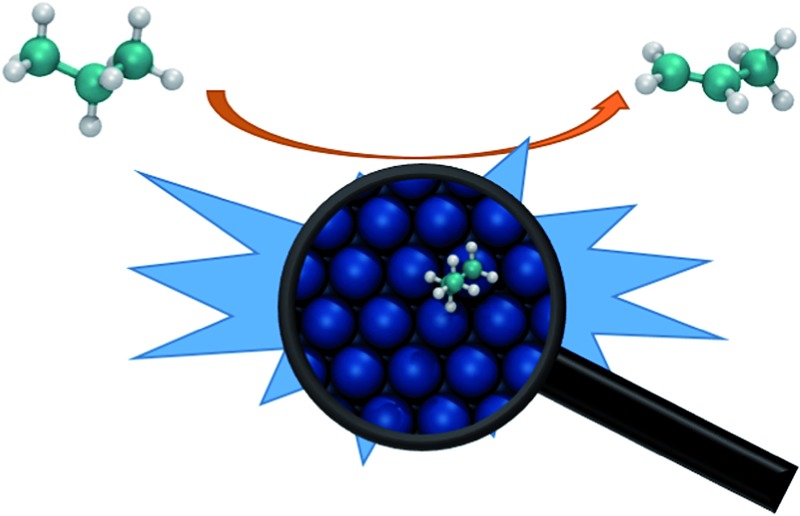
This review describes recent progress on mechanistic understanding of heterogeneous catalytic dehydrogenation reactions of light alkanes.

## 1 Introduction

Activation of hydrocarbons over heterogeneous catalysts is a dynamic and growing field of research since the birth of the modern petroleum and natural gas industry. Recent development of hydraulic fracturing or “fracking” technologies have shown the ability to efficiently extract shale gas, which will increase the supply of CH_4_ as well as other light alkanes, mainly ethane and propane,^1^ rendering them a cheap and reliable source for chemical industry.^2^ On the other hand, light alkenes are important feedstocks for the production of polymers, oxygenates and many other important chemical intermediates. Currently, the most common approaches to produce light alkenes are steam creaking and fluid catalytic cracking (FCC) of naphtha, light diesel and other oil byproducts.^1^ In 2007, 95% of propylene was produced as a byproduct of ethylene plants and other refineries.^3^ Although the recovery rate of propylene from a FCC unit have increased from 29% in 1980 to ∼80% in 2009, the fast growing demand for propylene still pushes up its price.^4^ Thus, an alternative process, such as the propane dehydrogenation (PDH), shows a high potential in profitability due to the large price difference between propane and propylene.

Currently, the most commonly used commercial PDH catalysts include supported Pt or CrO_*x*_. Although supported Pt catalysts show high activity, the catalyst deactivation due to coke formation remains a challenging issue.^5^ Moreover, since PDH is an endothermic reaction (Δ*H*_298 K_ = 124 kJ mol^–1^), high reaction temperatures favor transformation from propane to propylene. However, the undesired deep dehydrogenation leading to coke formation is also enhanced at high temperatures. Thus, the development of strategies to avoid coke formation is an important field of research. The robustness against coking can be increased with additional promoters, such as late transition metals, main-group metals including Sn and Ga, alkali-metal oxides and rare-earth metal oxides.^6^ However, the nature of atomic structure of these promoters during the dehydrogenation process is still under debate.

Besides the production of propylene, the activation of the C–H bond in methane is also an important but challenging process. The relatively stable methane C–H σ bond, the negative electron affinity, the large ionization energy, the absence of a dipole moment and the extremely high p*K*_a_ renders methane highly resistant to attacks by most redox active reagents, acids and bases. One of the most widely used technologies is the reforming process. It converts methane as well as other hydrocarbons into synthesis gas or hydrogen (with other byproducts), which can be further used in various processes to yield value added chemicals. Although this process has been extensively studied for nearly half a century, researchers are still trying to gain more insights into the reforming process by means of modern (*in situ*) characterization techniques, as well as theoretical calculations.^7^

**Fig. 1:**
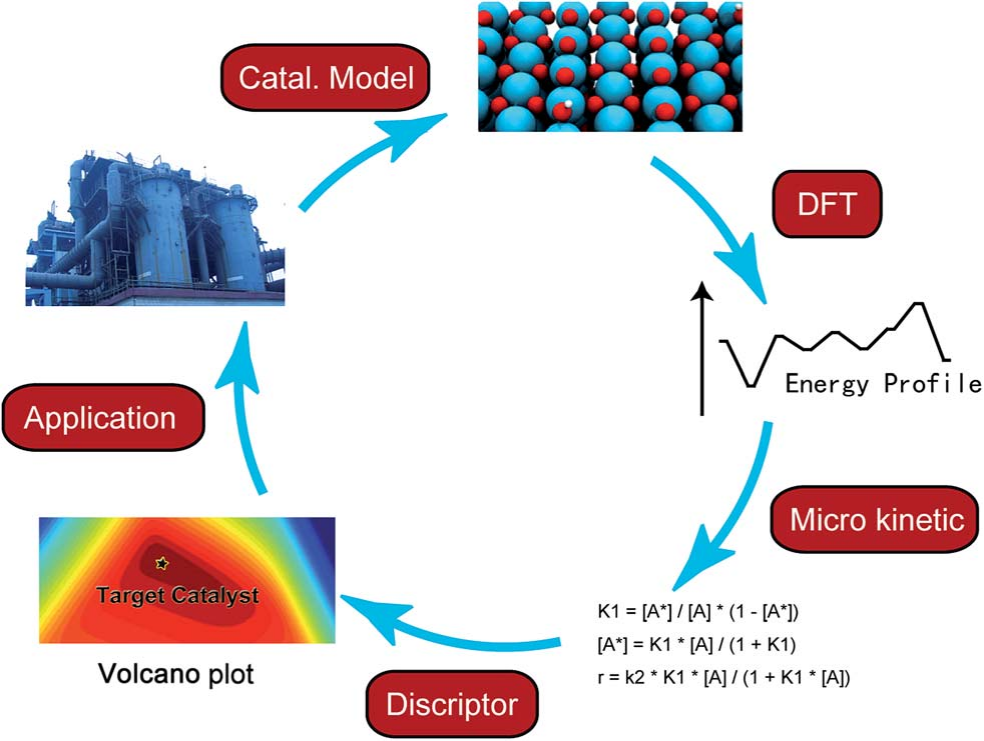
Scheme of computer aided catalyst design.

In the last few decades, density functional theory (DFT) has become a powerful tool for studying heterogeneous catalytic processes and their elementary steps and mechanisms at atomic scale. Such an “atomic resolution” of the processes is very hard to be achieved experimentally. Along with the development of novel computational architectures and the exponential increase in computational processor speed, the catalytic systems treated by DFT have developed from simple models such as metal or oxide single crystal surfaces to more sophisticated ones including alloys, supported catalysts and zeolites *etc.* In addition, with advanced kinetic modeling, the rate constants of elementary steps calculated with DFT can be further converted to turnover rate under reaction conditions. By applying linear correlations between activation energies and descriptors, *e.g.* binding energy or Brønsted–Evans–Polanyi (BEP) relationship, the activity and selectivity of a chemical process can be deduced from a small number of descriptors instead of from a large amount of detailed, but also hard to overview, information of all elementary steps. As the reaction rates are essentially governed by only a few descriptors, a large number of potential catalysts can be screened rapidly by means of computational chemistry.^8,9^ Nevertheless, the fundamental understanding offered by DFT calculations still serves as a cornerstone in the complete process of computer-aided catalytic design (Fig. 1).

This review describes mechanistic insights into heterogeneous catalytic dehydrogenation of light alkanes obtained from DFT calculations. The progress of dehydrogenation using homogenous catalysts was recently reviewed by Balcells *et al.* ^10^ and it will not be included in this review. We start with the background of density functional theory and its application for heterogonous catalysts. We then provide an overview addressing dehydrogenation reactions on metal or alloy surfaces, which represent the most studied area under this topic. The reactions on three other types of catalysts, including oxides, zeolites and singe site (atom) catalysts are covered in the Section 4. We then turn to the review of mechanistic studies on coke formation, which is one of the most common factors leading to catalyst deactivation during dehydrogenation reactions. A summary and outlook is given in the last section.

## 2 Background of DFT calculations in heterogonous catalysis

For the investigation of solid states, DFT is an important and powerful method for quantum mechanical modeling developed in the last century. The origin of DFT can be traced back to the Thomas–Fermi model in 1920s.^11,12^ From the 1960s, the formulation of DFT addressed by Kohn and Sham (KS), became the basis of current routinely used computational methods.^13^ In the KS formalism, the entire unknown part of the energy functional is collected in the exchange–correlation energy *E*_XC_, where approximations are used. The approximation can have different levels of sophistication, which is compared by Perdew and Schmidt to the rungs of the “Jacob's ladder” of density functional approximations.^14^ Nowadays, most popular functionals used in periodic systems are mainly at the second rung, *i.e.* generalized gradient approximation (GGA) level, for example Perdew–Wang 1991^15^ (PW91) and Perdew–Burke–Ernzerhof^16^ (PBE) functionals, which feature a high accuracy-to-cost ratio for many applications. However, for studies on heterogeneous catalysts, both PW91 and PBE functionals have several shortcomings, including over binding of surface intermediates^17^ and lack of van der Waals (vdW) dispersion interactions.^18^ The later developed revised PBE functional (RPBE^17^) improves the reproducibility of experimental binding energies for surface species. Recently, more attempts have been done in order to account for vdW dispersion between non-overlapped densities with several different approaches, and a new family of “vdW” functionals, such as vdW-DF,^19^ BEEF-vdW^20^ *etc.*, have been developed. The performance of functionals at different levels have been compared in several test studies.^21,22^

To model a catalytic particle/surface, the simplest and most common way is to employ a slab model of a low Miller index surface, with typically 3–6 atomic layers thickness in case of modeling metals. The terrace, edge and corner atoms on a metal particle can be approximately described by corresponding atoms on flat, step and kink surfaces, respectively. However, if the studied catalyst consists of ultra-small nanoparticles or sub-nanometer clusters, a non-periodical cluster model may be more reasonable due to the existence of quantum size effects.^23^ Recent researches start to take the support into account, using either a metal cluster/particle,^24^ an 1-D periodic nanowire,^25^ or a 2-D periodic catalyst slab supported on a 2-D periodic support slab. The former two types of models, *i.e.* supported clusters or nanowires, explicitly contain the sites at metal/support interface, which may be essential for the understanding of the catalytic activity at the catalyst–support interface of a bi-functional catalyst.

To describe a reaction, at least three data points need to be located on the potential energy surface: the initial, transition and final states. The optimization of initial and final states are relatively simple. However, the identification of a transition-state structure, which is a saddle point on the potential energy surface, is more challenging and computationally expensive. Moreover, due to the lack of an analytical second derivative for plane wave based DFT code, so-called mode-following methods which find transition states by following low curvature directions from energy minima are not easily implemented.^26^ The most widely used methods in this area can be categorized into two groups: (1) Elastic Band (EB) method and its improvements such as the Nudged Elastic Band method (NEB) and the climbing image nudged elastic band method;^27^ (2) dimer method.^28^ The EB family of methods locate a transition state by simultaneously optimizing a chain of structures between the initial and final states, which are connected by an “elastic band”. In the dimer method, the algorithm moves two images (dimer) uphill along the lowest curvature mode, which is estimated according to Voter's hyperdynamics method without evaluating the Hessian matrix, on the potential energy surface.^28^ Because of the complexity associated with the identification of a transition state, other attempts have been made to estimate the reaction barrier without explicitly obtaining the transition state structure and energy. Widely used ones include the BEP relationship^29^ and other types of scaling relationship. All of these attempts try to obtain linear correlations between activation barriers and other easily calculated quantities, or so called descriptors, such as reaction energies,^29^ d-band center of metal slabs,^30^ and atomic binding energies.^31^

The energetics obtained directly from DFT calculations describe the potential energy surface at 0 K and 0 bar. To describe a chemical process under realistic reaction conditions, thermodynamic corrections are necessary, which includes the zero point energy as well as the contributions to enthalpy and entropy at higher temperature and pressure. These corrections can be easily calculated according to statistical thermodynamics for an ideal gas. However, the corrections for an adsorbed system, especially for the soft frustrated translation and rotation modes, are not always well defined. Unfortunately, these soft modes have a large contribution to the total entropy. Thus, they cannot be completely ignored. Several different approaches have been made to estimate this entropy, including treating soft modes in the same way as other vibration modes,^32^ employing a harmonic well model,^33^ or estimating from gas-phase entropy values.^34^

Although the calculated reaction energies and barriers of the elementary steps provide useful clues to interpret the reaction mechanism, a more reliable method is to solve the steady-state at reaction conditions based on the calculated kinetic and thermodynamic information. Two common approaches include micro-kinetic modeling, which uses a mean-filed description,^33^ and kinetic Monte Carlo (kMC) simulation which includes the explicit consideration of the correlations, fluctuations and spatial distributions of the adsorbates at the catalyst surface.^35^ By including the linear relationship between barriers and descriptors mentioned above, the activity and selectivity predicated by kinetic analysis can be projected from a high dimension space including information of all elementary steps to a simple descriptor space, and a volcano plot can be generated for a direct guide to locate the optimal catalyst.^36^

## 3 Metal surfaces

### 3.1 Pure metal surfaces

#### 3.1.1 Methane activation

As the simplest hydrocarbon, activation of methane on transition-metal surfaces has been used numerous times as a model system since the early stage of theoretical calculations. As early as 1996, Kratzer *et al.* ^37^ reported a barrier at 108 kJ mol^–1^ for the first dehydrogenation step on Ni(111), which is one of the most widely calculated metals for methane decomposition due to its application in the steam methane reforming process.^38^ Till now, the activation of methane is still a hot topic, owing to the recent shale gas boom. Although the steam reforming process is now widely used in the modern chemical industry, other alternative routes to process methane are still quite challenging. Such alternative processes include, among others, the direct conversion of methane to other valuable chemicals or the dry CO_2_ reforming which not only reduces greenhouse emission but also produces a H_2_/CO mixture with a H_2_/CO ratio adequate for the Fischer–Tropsch process.^39^ Understanding the activation mechanism of the C–H bond in methane serves as the first step to tackle the nature of various processes for methane conversion. In the following, we denote, for convenience, the *x*th dehydrogenation step as D*x*.

##### Methane dehydrogenation on fcc(111) and hcp(0001)

**Fig. 2:**
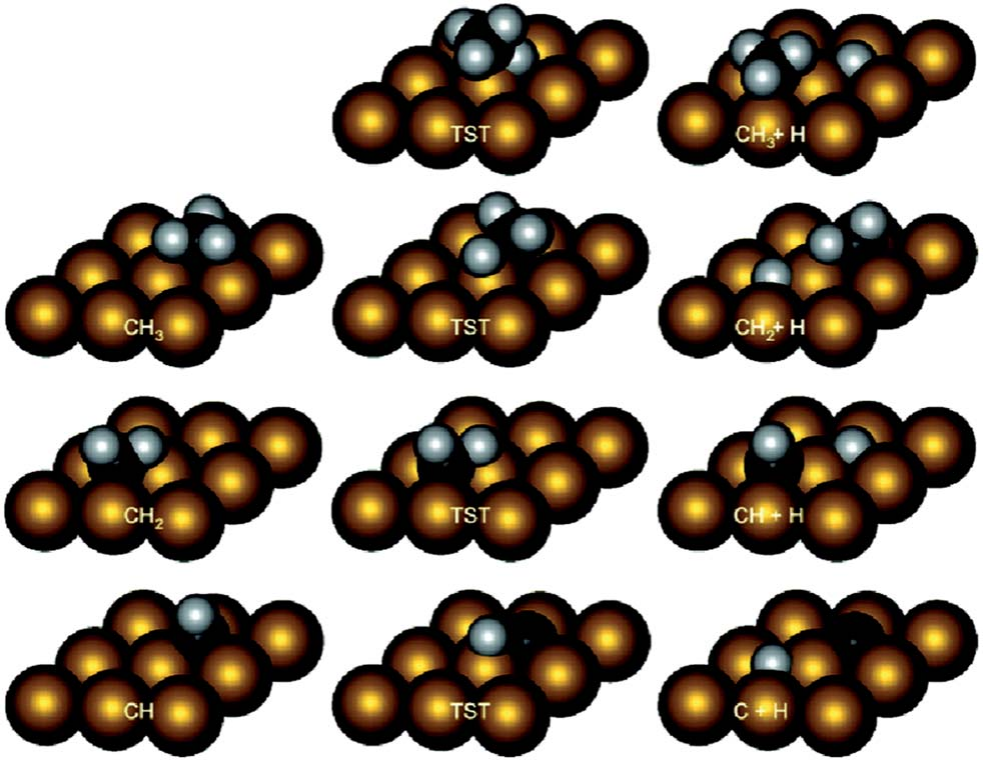
Intermediate and transition state structures of CH_4_ dehydrogenation on Rh(111). Adapted with permission from ref. 52. Copyright 2011 American Chemical Society.

Table 1 collected parts of previously calculated reaction energies and barriers of CH_4_ decomposition steps over close packed fcc(111)^40–60^ or hcp(0001)^61^ surfaces. The most obverse signature for this series of reactions is the relatively high barriers for the first (D1) and last (D4) dehydrogenation steps, among many of which the barriers are higher than 100 kJ mol^–1^ (Table 1). In contrast, the barriers for D2 and D3 steps are mostly less than 80 kJ mol^–1^, and can be even as low as <10 kJ mol^–1^. A necessary condition to form such a trend is the similar transition states of these dehydrogenation reactions over hexagon-shaped fcc(111) or hcp(0001) surfaces: the C–H bond is activated by interacting simultaneously with a surface metal atom, forming C–M–H three-membered ring structure (Fig. 2).

**Table 1:** List of calculated reaction and activation energies (kJ mol^–1^) for methane dehydrogenation on different metal surfaces. *E*(s): reaction energy with infinite separation of products; *E*(c): reaction energy with co-adsorbed species; *E*_a_: activation energy

	D1			D2			D3			D4									
Surface	CH_4_ = CH_3_ + H			CH_3_ = CH_2_ + H			CH_2_ = CH + H			CH = C + H			XC	Cut-off/eV	*k* points	Unit cell	No. layers^*d*^	Software	Ref.
	*E*(s)	*E*(c)	*E*_a_	*E*(s)	*E*(c)	*E*_a_	*E*(s)	*E*(c)	*E*_a_	*E*(s)	*E*(c)	*E*_a_							
Ni(111)		88	126		15	86		–37	40		41	133	RPBE		15	2 × 2	1r 2f	ADF-BAND	46
Ni(100)		65	119		9	60		–32	21		–3	62	RPBE		15	2 × 2	1r 2f	ADF-BAND	46
Ni(553)		8	104		–8	69		–49	14		–28	45	RPBR		12	2 × 1	5r 8f	ADF-BAND	46
Ni(111)	38		114	20		75	–33		36	59		131	PBE	340	5 × 5 × 1	2 × 2	2r 1f	CASTEP	40
Ni(111)				5	34	77	–43	–27	35	49	81	137	PBE	DZP^*a*^	5 × 5 × 1		1r 3f	SeqQuest	41
Ni(111)	–1^*c*^		88	7^*c*^		68	–33^*c*^		34	50^*c*^		128	PBE	400	3 × 3 × 1	3 × 3	3r 1f	VASP	42
Co(111)	7	31	104	17	42	60	–38	25	39	35	107	119	PW91	340	5 × 5 × 1	2 × 2	3r 1f	CASTEP	45
Cu(100)	70^*c*^		145	65^*c*^		116	33^*c*^		72	98^*c*^		170	PBE	DZP^*a*^	2 × 2 × 1	4 × 4		SIESTA	47
Cu(111)	76	86	171	82	92	148	56	61	109	143	144	191	PBE	DZP^*a*^	4 × 4 × 1	3 × 3	2r 2f	Dmol	50
Cu(100)	87	84	153	81	104	150	12	26	74	84	86	149	PBE	DZP^*a*^	4 × 4 × 1	3 × 3	2r 3f	Dmol	50
Cu(111)	72		151	80		131	40		91	118		178	PBE	400	5 × 5 × 1	3 × 3	2r 2f	VASP	48
Cu(111)	95		158			142	49		100				PBE	400	7 × 7 × 1	2 × 2	2r 2f	VASP	48
Cu(111)	96		188	92		138	46		117	130		205	PBE	340	5 × 5 × 1	2 × 2	3f	VASP	49
Cu(211)	33		138	79		134	13		184	75		176	PBE	340	4 × 4 × 1	3 × 3	3f	VASP	49
Ru(0001)^*b*^	–6	15	85	–5	18	49	–45	–33	16	35	59	108	PW91		5 × 5 × 1	2 × 2	4r	VASP	61
Rh(111)	11		79	–10		53	–50		6	54		108	PW91	310	5 × 5 × 1	2 × 2	3r 1f	CASTEP	51
Rh(100)	–13		62	–10		32	–60		5	–22		68	PW91	310	5 × 5 × 2	2 × 2	3r 1f	CASTEP	51
Rh(110)	–2		67	–11		30	–12		111	–17		50	PW91	310	3 × 5 × 2	2 × 2	3r 1f	CASTEP	51
Rh(111)	–49	–7	58	–26	–62	53	–95	–63	3	46	36	104	PW91	400	3 × 3 × 1	3 × 3	5	VASP	52
Rh(211)	–87	–40	35	–55	–12	60	–112	–63	25	–16	24	78	PW91	400	3 × 3 × 1		6	VASP	52
Rh(111)	10	21	67		10	41							PBE	367	4 × 4 × 1	2 × 2	3r 1f	QESPRESSO	53
Rh(111)^*b*^	9	29	70			47			10				PW91	400	5 × 5 × 1	2 × 2	2r 1f 2r		54
Pd(111)		35	92		21	100		–20	57		99	120	PW91	400	4 × 4 × 1	2 × 2	4	CASTEP	60
Ir(111)	–30	11	90	–34	–19	48	–53	–71	39		75	149	PBE	380	6 × 6 × 1	2 × 2	2r 2f	CASTEP	56
Pt(111)	–13	5	82	33	5	97	–38	–58	57	84	61	149	PBE	380	6 × 6 × 1	2 × 2	1r 2f	CASTEP	56
Pt(111)	–28^*c*^		61	7^*c*^		80	–63^*c*^		16	45^*c*^		124	PBE	DZP^*a*^	5 × 5 × 1	2 × 2	2r 2f	SIESTA	62
Pt(211)	–55^*c*^		20	–44^*c*^		17	–43^*c*^		53	32^*c*^		124	PBE	DZP^*a*^	3 × 4 × 1	2 × 1	6r 6f	SIESTA	62
Pt(111)	21		90	7		72	74		16	24		120	RPBE	415	6 × 6 × 1	3 × 3	6	VASP	59

^*a*^Double-*ζ* plus polarization.

^*b*^Ads on both sides.

^*c*^Unspecified *E*(s) or *E*(a).

^*d*^ *x*r *y*f: *x* relaxed layer with *y* fixed layer

At least two factors contribute to the relative high barriers of D4 among all four dehydrogenation steps, electronically and geometrically. The electronic effect is influenced by the relative stability of dehydrogenated intermediates. CH is the most stable surface intermediate during the dehydrogenation process.^29^ The following D4 step starting from CH is always strongly endothermic and leads to a high thermodynamic barrier (*i.e.* reaction energy), over 50 kJ mol^–1^, on many calculated surfaces. Meanwhile, geometrically the perpendicular nature of CH is not favorable for further dehydrogenation, where an energy penalty needs to be applied to bend the structure parallel to surface in the transition state (Fig. 2). Both effects tend to push up the barrier of D4 step. Similar strong bending of the adsorbate is also necessary for the D1 step, in order to move the C atom more close to the metal surface in the transition state.

**Fig. 3:**
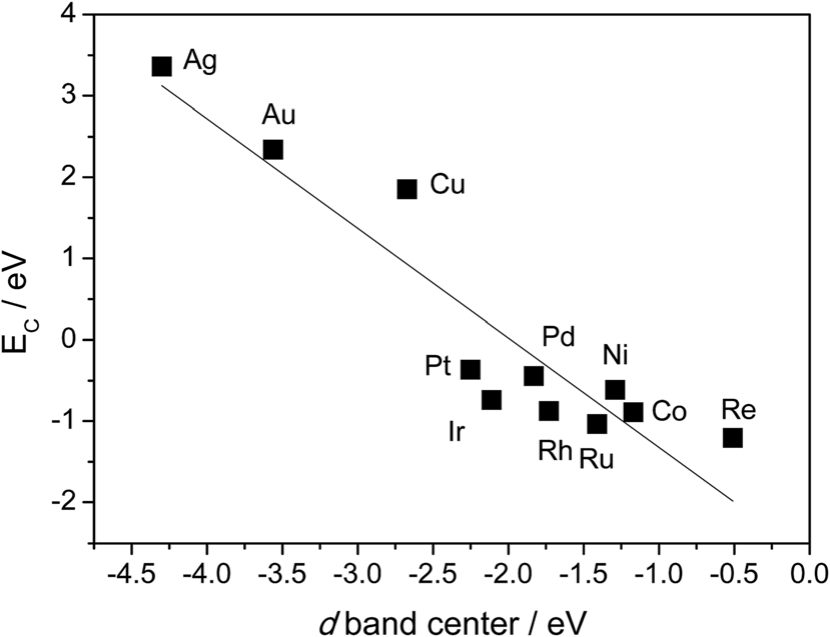
Relationship between C binding energy and metal d-band center. C binding energies are calculated with 3 × 3 unit cell, BEEF functional. Values of d-band center energies are adapted from ref. 63.

Table 1 also shows that, in general, CH_4_ decomposition barriers become higher along with the surface metal elements moving to the right of the periodic table. Following the Nilsson and Pettersson model,^62^ the interaction between doubly occupied orbitals of adsorbates and high electron occupied d orbitals is repulsive. As we move to the right of periodic table from Ru, there are more electrons filled in the metal d band, and the repulsion between occupied orbital interactions becomes stronger. This leads to weak binding of surface intermediates, as well as poor stabilization of transition states. Deeper dehydrogenation removes H atoms from the adsorbed intermediate, which results in more C–metal interaction, and eventually leads to higher reaction barriers. Indeed, the total thermodynamics for CH_4_ = C* + 4H* is exothermic by 21 kJ mol^–1^ on Ru(0001), where Ru has 7 electrons in the d band. However, on Cu(111) where Cu has a filled d band, this process becomes strongly endothermic by 364 kJ mol^–1^, which is even much higher than highest kinetic barrier over Ru(0001). It has been suggested that this effect can be simply characterized by the d-band center solely, where stronger binding of the adsorbate is expected in case of a metal with higher d-band center (Fig. 3).^63^

Another interesting observation is the high D1 barriers (>100 kJ mol^–1^) on 3d metals compared with the D1 barriers (<100 kJ mol^–1^) on 4d or 5d metals. For elements in the same column, the main difference between them is the extension of the d atomic orbitals.^29^ The 3d orbitals of Ni, Co and Cu are more contracted, while the transition metals in the fourth or fifth row are found with larger extension of the d atomic orbitals, which leads to preferential adsorption of the adsorbates such as CH_3_, CO or NH_3_ on top sites.^64^ This process minimizes the similar repulsion interaction,^29^ which results in the stronger binding and lower barrier for 4d and 5d series.

##### Methane dehydrogenation on fcc(100)

The (100) facet is a more open surface whose surface atoms have a coordination number (CN) of 8. On Ni(100), the rate determining step for CH_4_ decomposition is the D1 step, with >120 kJ mol^–1^ barrier. The barriers of the other three steps are <65 kJ mol^–1^. Interestingly, all four dehydrogenation barriers on Ni(100) are lower than the corresponding ones on Ni(111), which has been connected to the higher d-band center of Ni(100) surface (–1.64 eV) than of Ni(111) (–1.78 eV).^46^ Besides the electronic effect, a strong stabilization of adsorbed C atoms was observed on Ni(100), which binds >100 kJ mol^–1^ stronger than a C atom on Ni(111). The more stable tetra-coordinated C on Ni(100) reflects the needs of a C atom to satisfy its valence.^46^ It further results in that the D4 barrier is only 62 kJ mol^–1^ on Ni(100), much lower than the barrier on Ni(111), which is >120 kJ mol^–1^. Similar stabilization of adsorbed carbon atom and low barriers of D4 steps were also observed on Cu(100)^50,65^ and Rh(100).^51^

##### Methane dehydrogenation on stepped fcc(211) surfaces

Compared with barriers on terrace (111) surfaces, lower dehydrogenation barriers of D1 and D4 were reported on stepped (211) surfaces.^49,50,66^ The first clue related to this observation is the existence of low-coordinated step atoms on (211) surfaces. Since the binding strength of surface intermediates tends to increase along with the decreasing of coordination number,^32^ there is more stabilization of transition and final states of D1 step at low coordinated step site of (211) than on corresponding (111) surfaces. However, the initial state of this reaction is physically adsorbed CH_4_ whose binding strength is not influenced by the coordination number of surface atoms. Thus, D1 barriers on the (211) step edge are lower than the corresponding ones on the (111) terrace site. In the following D2–D4 dehydrogenation steps, all the initial, transition and final states have already bound to edge sites, and the additional stabilization between the low coordinated edge atoms and adsorbed intermediates mostly cancels out. In addition, the geometry effect also influences the relative barrier heights of these steps on step and terrace surfaces. Additional bending in the transition states of D3 step on Rh(211)^50^ and Cu(211)^49^ induces higher D3 barriers on (211) surfaces than the corresponding barriers on (111). While reverse effects were observed for the D4 step, resulting in lower barriers on (211) surfaces.^49,50^

##### Methane steam reforming on transition-metal surfaces

**Fig. 4:**
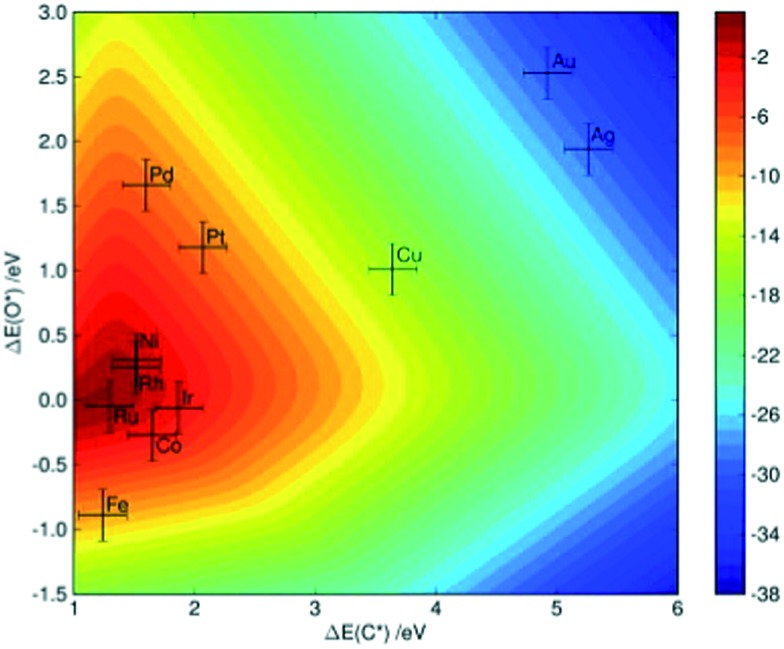
Two-dimensional volcano-curve of the turnover frequency (log_10_, at 773 K, 1 bar) as a function of C and O binding energy. Reprinted with permission from ref. 7. Copyright 2008 Elsevier.

Jones *et al.* ^7^ performed a detailed kinetic analysis on the methane steam reforming reactions over more than 10 transition-metal surfaces. By applying linear scaling relationships between the binding energies of selected molecular fragments, which serve as descriptors, and the binding energies of intermediates as well as of transition states that occur in the reforming process, the reaction rate (as characterized by the turnover frequency) can be simply mapped to a two-dimensional descriptor space, which is defined by the binding energy of C and O in that study. As indicated in Fig. 4, theoretical calculations predict that the steam reforming activity decreases in the sequence Ru > Rh > Ni > Ir > Pt ≈ Pd, which is comparable with experimental observations showing Ru ≈ Rh > Ni ≈ Ir ≈ Pt ≈ Pd.^7^ The small discrepancies might arise due to the different morphology of the nanoparticles under experimental conditions, as well as due the uncertainty of DFT calculations and the errors introduced by the scaling scheme.

#### 3.1.2 Ethylene dehydrogenation

Ethylene transformation is one of the most widely studied model system to understand hydrogenation/dehydrogenation reactions. On Pt(111), ethylene is known to form two types of adsorption complexes: π-adsorbed ethylene has been observed at very low temperatures or in coadsorbed systems whereas a transformation to a di-σ bonded species begins upon heating above 52 K.^67^ Further heating of the surface will lead to conversion to ethylidyne, CH_3_–C≡, on many transition-metal surfaces such as Pt(111),^68^ Rh(111),^69^ Pd(111),^70^ Ir(111)^71^ and Ru(0001).^72^ Although the transformation from ethylene to ethylidyne seems simple with only one H atom removed from hydrocarbon, the mechanism of this transformation has long been debated. Based on the extensive kinetic and spectroscopic studies, Zaera and French^73^ suggested a two-step mechanism of ethylidyne formation: a direct 1,2-H shift reaction converts ethylene to ethylidene (CH_3_CH), followed by a dehydrogenation reaction to form ethylidyne.

However, a later theoretical study^74^ provided strong evidence against the two-step mechanism. The calculated 1,2-H-shift barrier from ethylene to ethylidene was as high as ∼200 kJ mol^–1^ on Pt(111). Similar high barriers for H shift reactions were also observed on Pt(110),^75^ Pt(211),^58^ Pd(111),^76^ Rh(111)^77^ and Fe(100).^78^ Although the substrate metals or surface structures are different from each other, a common observation for this type of H-shift reaction is a C–H–C three membered ring transition-state structure. The C–H–C ring does not directly interact with surface metal atoms, indicating little assistance of the metal catalyst in stabilizing the transition state during this conversion.

**Fig. 5:**
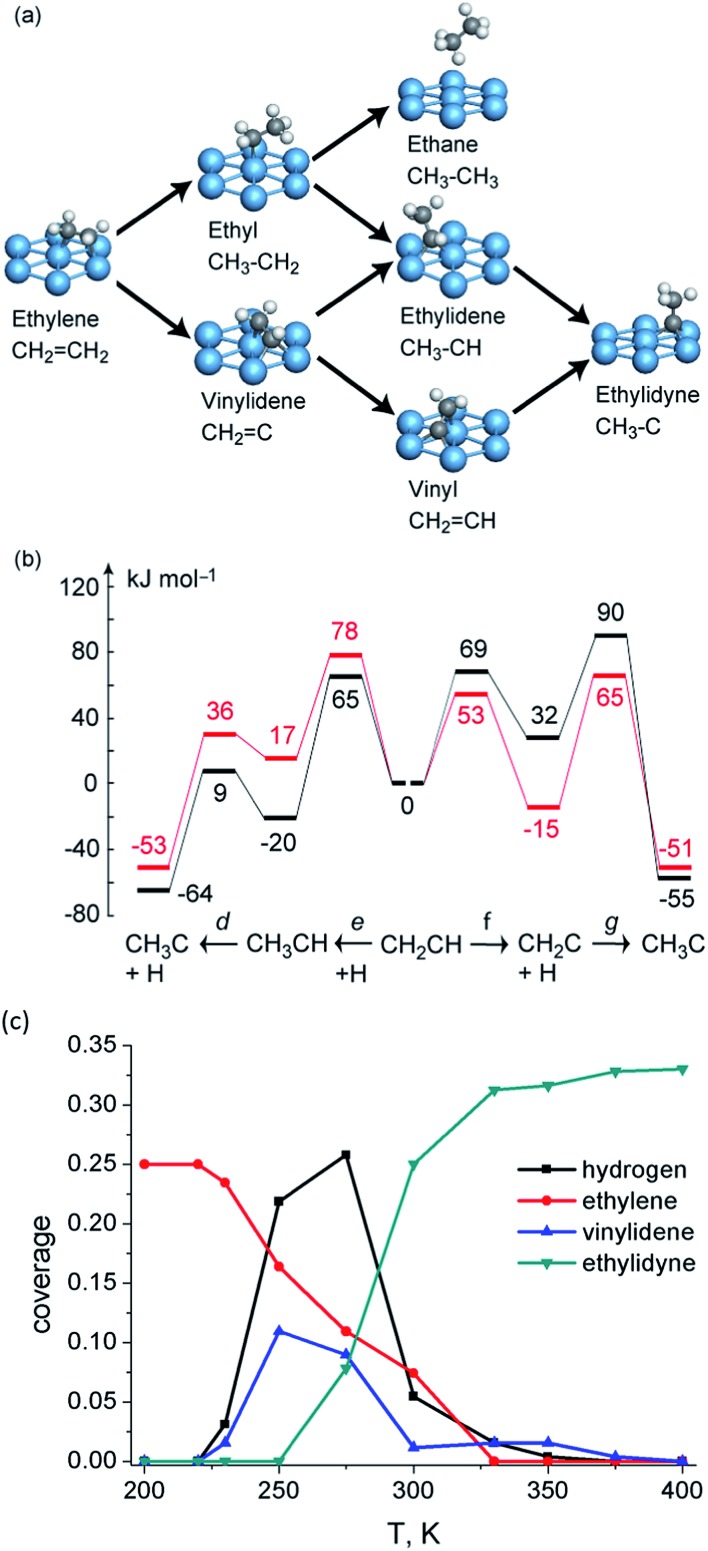
(a) Reaction network of ethylene transformation over Pt(111); (b) reaction energy profiles (kJ mol^–1^) of vinyl conversion to ethylidyne over Pt(111) at 1/3 coverage (black) and 1/9 coverage (red). Reprinted with permission from ref. 74. Copyright 2010 American Chemical Society; (c) kMC simulated surface coverage after 1 s reaction over Pt(111). Reprinted with permission from ref. 79. Copyright 2012 Elsevier.

Even if the hydrogen shift reactions have been excluded due to their high barrier, there are still at least three competing pathways for this conversion: (1) CH_2_CH_2_ → CH_2_CH → CH_3_CH → CH_3_C; (2) CH_2_CH_2_ → CH_2_CH → CH_2_C → CH_3_C; (3) CH_2_CH_2_ → CH_3_CH_2_ → CH_3_CH → CH_3_C (Fig. 5a). DFT calculations indicate^74^ that at low coverage (1/9 ML), the conversion prefers a mechanism *via* two consecutive dehydrogenation steps to form CH_2_CH and then CH_2_C, with a final hydrogenation step to form CH_3_C (Fig. 5b). When the coverage is increased to 1/3 ML, the dehydrogenation barrier increases in general by ∼20 kJ mol^–1^.^74^ The dominated mechanism still starts from dehydrogenation to CH_2_CH, but shifts to hydrogenation reaction in the second step to form CH_3_CH and the last dehydrogenation to form CH_3_C (Fig. 5b).

Due to the existence of the coverage effect, it is not easy to identify dominating reaction mechanisms under reaction conditions purely based on DFT-calculated barriers. Further clarification of the mechanistic scenario is achieved by kMC simulations, which allow one to track explicitly the behavior of all surface species as a function of time and processing conditions. The kMC study^79^ predicts the conversion of ethylene starts at temperatures as low as 230 K on Pt(111). The dominated mechanism predicted by kMC follows the route (2) CH_2_CH_2_ → CH_2_CH → CH_2_C → CH_3_C. The third hydrogenation step is rate limiting, which results in the accumulation of CH_2_C on surface. Interestingly, in some cases, even with a lower hydrogenation barrier, these hydrogenation steps can still be slower than dehydrogenation steps with a higher barrier due to the limited amount of adsorbed hydrogen atoms.

Indeed, experimentalists observed an intermediate during ethylene conversion on Pt(111). Spectroscopic studies with sum frequency generation (SFG)^80^ and reflection adsorption inferred spectroscope (RAIRS)^81^ methods observed a peak at ∼2960 cm^–1^ and assign it to asymmetric stretching of the CH_3_ group in CH_3_CH. A second peak at 1387 cm^–1^ was observed which developed in parallel with the 2960 cm^–1^ feature by the latter study and was assigned to the symmetric bending of ethylidene. Both assignments contradict with the very low DFT barrier for dehydrogenation of ethylidene as well as the kMC simulation results, which indicate accumulation of vinylidene instead of ethylidene during ethylene conversion. Further DFT calculations^74^ indicated that both vinylidene and ethylidene have modes which locate close to 2960 and 1387 cm^–1^ due to the structure similarity of these C_2_ intermediates. Moreover, with deuterium substituted spectra, the C–C stretching mode of vinylidene is separated by at least 200 cm^–1^ with modes belonging to ethylidene, suggesting a possible way to identify this puzzling intermediate by future experiment.^82^

The formation mechanism of ethylidyne has also been studied on Pd(111)^79,83,84^ and Rh(111).^77^ On both surfaces, kinetic analyses predict that the dominant reaction mechanism is *via* the vinyl and vinylidene route, which is the same as on Pt(111). In general, the hydrogenation/dehydrogenation barrier heights follow the trend Rh(111) < Pt(111) < Pd(111),^85^ which can again be qualitatively explained by the filling of the d band, as discussed in methane decomposition (Section 3.1).

The binding of surface intermediates tend to be stronger on (211) surfaces than on the corresponding terrace (111),^58,76^ due to the low coordinated nature of step edge atoms. Besides the enhanced binding, CH_3_CH dehydrogenation barriers on (211) surfaces are significantly higher (*e.g.*, >80 kJ mol^–1^) than the corresponding barriers (<40 kJ mol^–1^) on (111) surfaces. By checking the transition-state structures, one can notice that this reaction is mainly catalyzed by a terrace metal atom, which is in the second row away from the step edge, although the reactant CH_3_CH and product CH_3_C directly binds to step edge. This leads to similar absolute energy of the transition state on both (111) and (211) surfaces. Thus, the barrier is higher on (211) because the initial CH_3_CH binds much more strongly at the step edge compared to on (111).

##### C–C bond breaking *vs.* dehydrogenation

As discussed in the ethylene conversion to ethylidyne section, only hydrogenation/dehydrogenation occur at low temperature range, *e.g.* below 400 K on Pt(111). Ethylidyne can be further dehydrogenated and coke can be formed at higher temperatures.^86^ Chen and Vlachos calculated all the possible C–C scission barriers from different C_2_ species on Pt(111) and Pt(211). On flat (111), most of the C–C bond breaking barriers are higher than 150 kJ mol^–1^, except for three cases, CC breaking from CH_3_CH (*E*_a_ = 114 kJ mol^–1^), CHCH (*E*_a_ = 103 kJ mol^–1^) and CHC (*E*_a_ = 88 kJ mol^–1^). Although the C–C breaking from CHC has a low barrier at 88 kJ mol^–1^, it was still proposed that C–C bond breaking might not occur *via* this pathway due to strong endothermicity of this step and the high formation barrier of CHC (>200 kJ mol^–1^). Instead, CHCH or CH_3_CH are more likely to be the precursor for C–C bond breaking on Pt(111). Since the highest dehydrogenation barrier during ethylene conversion to ethylidyne is below 100 kJ mol^–1^, the higher C–C bond breaking barriers prevent C_2_ cracking at low temperatures, which is consistent with the fact that C–C scission reaction can only occur over 540 K.^87^ Stepped Pt(211) in general shifts down C–C scission barriers from light dehydrogenated intermediates, and shifts up the ones from deep dehydrogenated intermediates. It is more likely that CHCH to be precursor of C–C bond scission, with the barrier at 123 kJ mol^–1^. Although the lowest C–C breaking barrier is from CH_3_CH_2_, it might not be the precursor because of the lack of surface H, particularly after CH_3_C is formed prior to the C–C bond breaking.

**Fig. 6:**
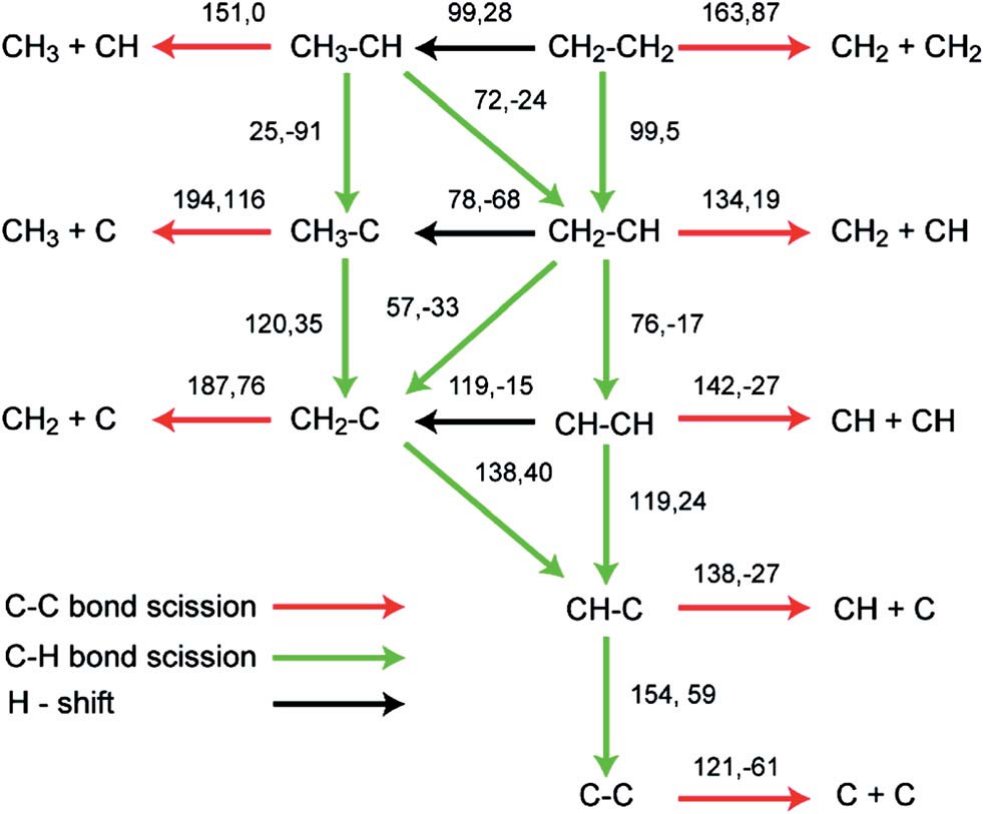
Reaction barriers and reaction energies (black, first and second values, respectively; in kJ mol^–1^) for various C–H and C–C bond-breaking reactions of ethylene and related species C_2_H_*x*_ (*x* = 0–4) over a Pd(111) surface. Reprinted with permission from ref. 88. Copyright 2010 American Chemical Society.

As shown in Fig. 6, the lowest C–C bond breaking barrier on Pd(111) is 122 kJ mol^–1^ and the corresponding precursor is the C_2_ dimer.^88^ However, the formation of the C_2_ dimer suffers from a high dehydrogenation barrier (154 kJ mol^–1^) from CHC. Hence, Chen *et al.* suggested^88^ that the formation of C_1_ species is most probably *via* a CH_2_C → CHC → CH + C pathway, where the precursor to form CH_2_C can be CH_3_C or CH_2_CH_2_ depending on the reaction conditions. The calculated CH–C dissociation barrier is 138 kJ mol^–1^, higher than the dehydrogenation barrier to form CHC, indicating possible accumulation of CHC during this conversion, which is in good agreement with experimental observations.^89^ A later study extended this network to Pd(211) surfaces, which again suggested the same CH_2_C → CHC → CH + C pathway for C–C scission. However, on stepped surfaces, rate determining step is CH_3_–C dehydrogenation to CH_2_C or C–C scission from CHC, whose barriers are both about 25 kJ mol^–1^ higher than dehydrogenation barrier from CH_2_C to CHC.

**Fig. 7:**
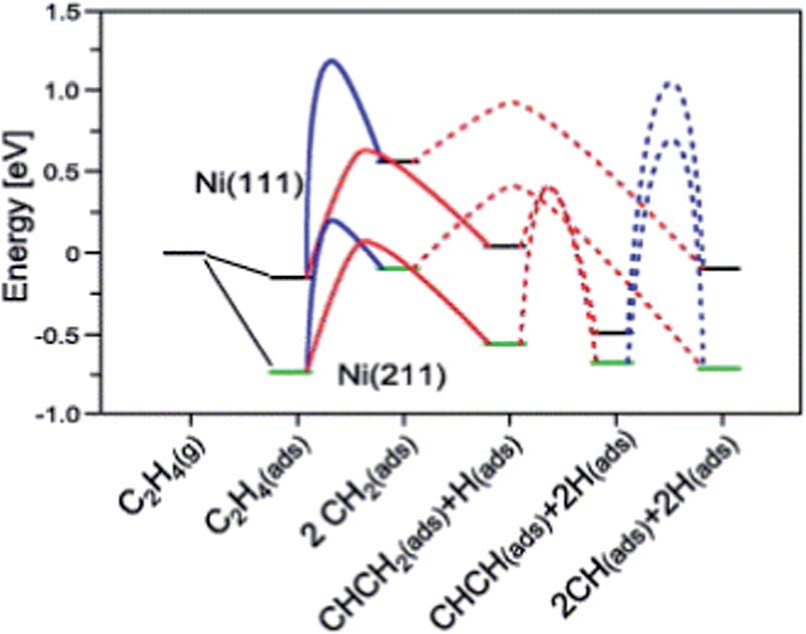
Potential energy diagram for C–C bond breaking (blue line) and C–H bond breaking (red line) on Ni(111) and Ni(211). The transition state energy for C–H bond breaking of CH_2_ is for one CH_2_. Reprinted with permission from ref. 90. Copyright 2006 Elsevier.

A simpler reaction network was employed by Vang *et al.* ^90^ to check the selectivity of ethylene dehydrogenation and cracking (C–C bond scission) on Ni surfaces (Fig. 7). It includes CH_2_CH_2_ dehydrogenations to CH_2_CH and CHCH, as well as C–C bond breaking from CH_2_CH_2_ and CHCH. On Ni(111), the initial dehydrogenation from ethylene is much more favorable than its cracking to form two CH_2_ groups, with about 50 kJ mol^–1^ lower dehydrogenation barrier. On stepped Ni(211), the ethylene cracking barrier dramatically decreases to about 100 kJ mol^–1^, which is comparable to its dehydrogenation barrier. The reduced barrier height of ethylene cracking, when comparing step sites with terrace sites, was explained by the geometry effect.^90^ The two CH_2_ groups, which are the final state of this cracking, locate on two threefold hollow sites on Ni(111). On Ni(211), one methylene adsorbs on a twofold sites, resulting in a much shorter distance between two CH_2_ groups than the pairs on Ni(111). It means that the transition state is stabilized at an earlier point in the former case, and thus the barrier of this reaction is lower on Ni(211). Nevertheless, step-edge sites are far more reactive towards ethylene decomposition than the regular sites on Ni(111), and thus play a very important role in the bond breaking selectivity between the initial C–H and C–C bond breaking.^90^

#### 3.1.3 Propane dehydrogenation

**Fig. 8:**
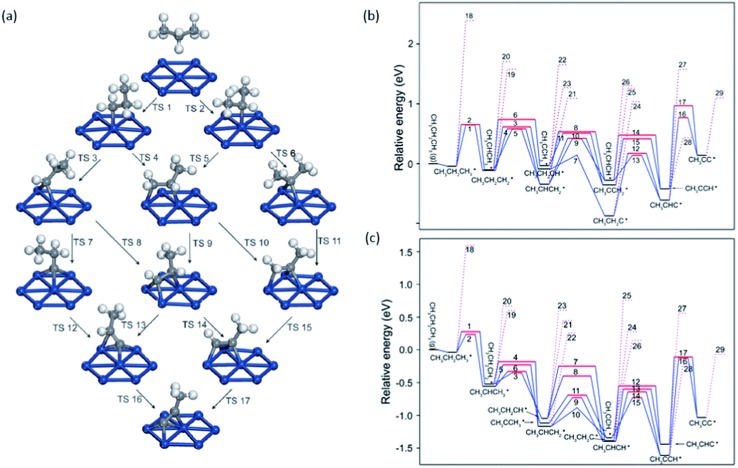
(a) Reaction network of propane dehydrogenation on Pt(111); (b and c) energy profile for propane dehydrogenation on Pt(111) (b) and Pt(211) (c) including both the dehydrogenation steps (solid lines) and the C–C cracking steps (dotted lines). Adapted with permission from ref. 92 with permission from the PCCP Owner Societies.

Propylene is one of the most important building blocks in chemical industries.^1^ Nowadays, it is dominantly produced by steam cracking of naphtha and fluidized catalytic cracking of heavy oil. Along with the decreasing fossil oil reserves and development on utilization of nature gas and shale gas, propane dehydrogenation (PDH) seems to be a promising alternative to produce propylene. Currently commercial PDH catalysts can be categorized into two groups: Cr- and Pt-based catalysts. The main problems of Pt-based catalyst are its low selectivity to propylene and fast deactivation caused by coke formation. Yang *et al.* ^91–93^ reported detailed studies of propane dehydrogenation on Pt(111), Pt(100) and Pt(211) surfaces, including 17 dehydrogenation steps (Fig. 8) and 11 C–C bond breaking steps. The dehydrogenation activity on these three surfaces follows the trend of Pt(211) > Pt(100) > Pt(111), based on the calculated dehydrogenation barriers. A stronger activity of the stepped surface for the dehydrogenation mechanism has also been reported by a work of Chiu *et al.* originally addressing the hydrogenation of propylene on the stepped Pt(221) surface.^94^ Since the desired product propylene is only partially dehydrogenated from propane, lower dehydrogenation barrier as well as stronger propylene binding will cause deep dehydrogenation and reduce the selectivity towards propylene. Yang *et al.* ^93^ also experimentally compared activity and selectivity of ∼12 nm cubic and octahedral Pt particles, which expose large area of Pt(100) and Pt(111), respectively. Although turnover frequencies (TOFs) showed that the cubic particles are more active than octahedral ones, higher propylene selectivity was observed on octahedral Pt with large surface area of Pt(111). On Pt(100), the binding energy of propylene is 118 kJ mol^–1^, which is 66 kJ mol^–1^ higher than its dehydrogenation barrier. The binding of propylene on Pt(111) decreases to 94 kJ mol^–1^, and the following dehydrogenation barrier increases to 73 kJ mol^–1^. Smaller barrier difference on Pt(111) indicates a stronger preference for propylene desorption on Pt(111) compared with Pt(100). Although desorption barriers are higher than the dehydrogenation barrier from propylene on both surfaces, the experimentally observed selectivities of propylene are still higher than 85% in both cases. The reason is that DFT results discussed here did not include thermodynamic correction, which would significantly reduce the free energy barrier for desorption steps due to the large entropy of gas phase propylene compared with the adsorbed ones.

#### 3.1.4 Methylcyclopentane dehydrogenation

**Fig. 9:**
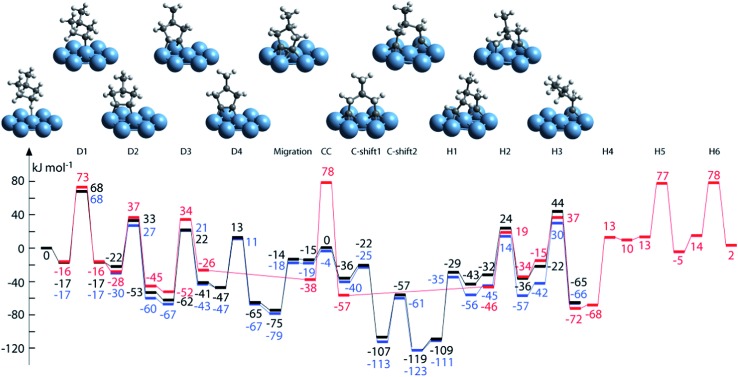
Structures and energy profiles of the MCP ring-opening reactions over Pt(111): reaction path to 2-methylpentane (2-MP) (black), 3-methylpentane (3-MP) (blue) and *n*Hx (red). Adapted with permission from ref. 95. Copyright 2012 Elsevier.

Cetane number is an important factor in determining the quality of diesel fuel. With the same number of C atoms, cetane number in general follows the rule of linear alkane > branched alkane > cycloalkane > aromatics. In order to increase the quality of diesel fuel, the aromatics can be saturated and subsequently cracked by advanced upgrading technologies, with preference to form more linear products and preserving the initial molecular weight. As a model selective-ring-opening reactions, ring-opening of methylcyclopentane on supported metal catalysts has extensively been studied. One interesting observation by previous studies is the particle size effect on Pt catalyst: large Pt particles prefer to produce branched pentanes, while small Pt particles equally break the endocyclic C–C bonds and generate a statistical distribution of branched and linear C_6_ products. DFT calculations^95,96^ attempted to model nanoparticles with different sizes by employing different types of surfaces, with Pt(111) to represent terrace-rich large particles and Pt(211) for the edge-rich small particles. The calculations indicate that a deep dehydrogenated precursor is necessary before the C–C bond breaking, which is similar as cracking of C_2_ and C_3_ hydrocarbons discussed above. The dehydrogenation and re-hydrogenation barriers, which are prior and after C–C bond breaking, respectively, are very similar to each other in all three ring-opening pathways (Fig. 9). However, transition states and barriers of the C–C bond breaking vary notably. In the reaction path to branched products on Pt(111), this barrier is about 75 kJ mol^–1^ *via* an ααββ-tetra-adsorbed cyclic intermediate. Due to the existence of the CH_3_ group attached to a dissociating C atom, only an ααβ-tri-adsorbed cyclic intermediate can be formed in the reaction path to linear *n*-hexane (*n*Hx). The corresponding C–C breaking barrier increases to 116 kJ mol^–1^ on Pt(111), which is at least 27 kJ mol^–1^ higher than the barriers of all other elementary steps, including those on the path to 2-MP and 3-MP (Fig. 9).^95^ Due to this high rate-limiting barrier, the formation of *n*Hx is suppressed on large Pt particles, which expose large surface area of Pt(111). However, the C–C cleavage barrier in the pathway to *n*Hx decreases to 79 kJ mol^–1^ at a step edge *via* an αγ-di-adsorbed intermediate.^96^ This barrier height is comparable to the hydrogenation/dehydrogenation steps, resulting in a statistical distribution of three products on small particles.

Similar analysis has further been done on Rh, Ir and Pd surfaces, with the assumption that deep dehydrogenation precedes ring cleavage.^97^ Based on the calculated barriers, the ring-opening activity follows the trend of Rh ≈ Ir > Pt > Pd, which agrees with experimental observations. The particle size effect on selectivity of MCP ring-opening products can also be rationalized by the calculated C–C bond breaking barriers. For example, the C–C breaking barrier leading to branched product is always lower on both Rh(111) and Rh(211) than barrier in the pathway to linear *n*Hx, which consists with experimentally observed favored production of branched methylpentanes.

### 3.2 Alloy surfaces

#### 3.2.1 Methane dehydrogenation on alloys

**Fig. 10:**
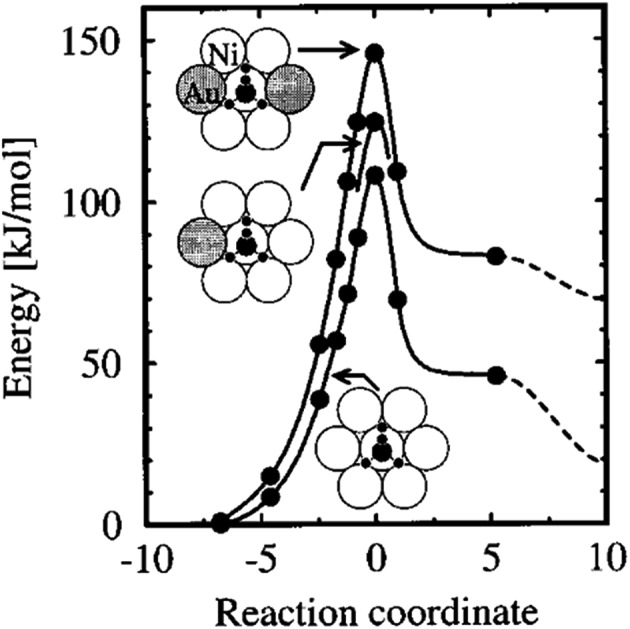
The calculated energy along the reaction path for CH_4_ dissociating over a Ni atom in the Au/Ni(111) surface. The rightmost data points (dashed curves) refer to infinite separation of the dissociated H and CH_3_ group on the surface. The dissociation geometry for the three chemical compositions is indicated by the insets, with gold atoms gray-shaded. Reprinted with permission from ref. 37. Copyright 1996 American Institute of Physics Publishing LLC.

One early attempt to probe the effect of alloying on the CH_4_ dissociation is on Au/Ni(111) by Kratzer *et al.* ^37^ In their model, one or two surface Ni atoms are replaced by Au atoms. Gold itself is unreactive with respect to CH_4_ dissociation, and blocks at least one active site by substitution of one surface Ni atom. Moreover, the existence of Au atoms also changes the electronic structure of neighboring Ni atoms, which leads to higher dissociation barriers, by 16 and 38 kJ mol^–1^ on a Ni atom with one and two gold neighbors, respectively, compared with the barrier on clean Ni(111) (Fig. 10). The existence of Au atoms shifts down the d band of alloys, which weakens the interaction between σ* of C–H bond and d states of surface metal atoms. Thus, the transition state gains less stabilization from surface atoms, and finally results in an increase of the dissociation barrier height. A similar study by Fan *et al.* ^43^ covers more X/Ni(111) surfaces, with X to be seven types of late transition metals, including Cu, Ru, Rh, Pd, Ag, Pt and Au. It clearly shows that a higher barrier on Ni_2_X site for all dehydrogenation steps in cases with less dehydrogenation-active metals, such as Cu, Ag and Au, embedded in Ni(111). In contrast, embedding a more dehydrogenation-active metal, *e.g.* Ru and Rh, tends to decrease the dehydrogenation barrier on Ni_2_X due to more empty d orbitals introduced by Ru and Rh. The last two metals, Pd and Pt, have similar dehydrogenation activity as Ni, and the dehydrogenation barriers on Ni_2_X site are similar to the values on Ni(111). On Ni_3_ sites without direct interaction with embedded X atom, the indirect electronic effect which is introduced by the embedded X atom shifts up most of dehydrogenation barriers, except for the case of Cu/Ni(111). In the latter case, all four dehydrogenation barriers are ∼5 kJ mol^–1^ lower than corresponding values on Ni(111). Note that this set of barriers are in disagreement with an earlier study An *et al.* ^98^ who reported the calculated barriers for all four dehydrogenation steps on Cu/Ni(111) are higher than the corresponding barriers on Ni(111).

In the case of a more reactive Rh atom embedded to a less reactive Cu(111) surface, the situation is slightly different. The C–Rh bond is roughly ∼50 kJ mol^–1^ stronger than C–Cu bond.^53^ In the first dehydrogenation step, the reaction can be catalyzed by the single Rh atom on the embedded Rh/Cu(111) surface. Thus, the calculated barrier of D1 on Rh/Cu(111), 68 kJ mol^–1^, is almost the same as the barrier on perfect Rh(111), 67 kJ mol^–1^. However, the subsequent dehydrogenation step generates methylene which binds to bridge sites, forming one C–Rh and one C–Cu bond. As expected, the D2 barrier on Rh/Cu(111), 81 kJ mol^–1^, is 40 kJ mol^–1^ higher than the barrier on Rh(111), because of the weaker binding of the product methylene on the anterior surface.

**Fig. 11:**
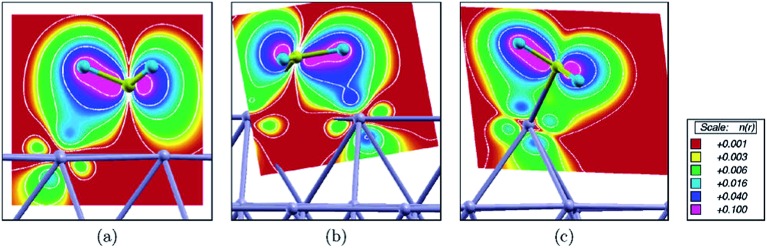
Integrated local density of states (ILDOS) illustrating the extent of three-center C–H–metal agostic bonding of methyl adsorbed on (a) a Rh(111) facet, (b) a step edge, and (c) an ad-atom. The magnitude of ILDOS increases from red to violet, following a rainbow scale. Five contours are drawn in logarithmic scale from 10^–1^ to 10^–3^ *e*/*a*_0_^3^. Reprinted with permission from ref. 53. Copyright 2006 American Chemical Society.

Besides the embedded model mentioned above, Kokalj *et al.* ^53^ further considered a case with a Rh as an ad-atom on hollow sites of Rh(111) as well as on Cu(111). The coordination number of the ad-atom is only 3, which is significantly smaller than CN of the surface atoms on (111) (CN = 9), (211) step edge (CN = 7) and ad-row atoms (CN = 5). It is expected that the strongest binding of a surface intermediate should be observed on an ad-atom due to its smallest coordination number. However, the calculated binding energy indicates that the strongest binding occur on ad-row atoms. The discrepancy was explained^53^ by the formation of an agostic bond, which is a three-center C–H–metal interaction normally with two electrons. Due to the three agostic bonds, fcc adsorbed eclipsed CH_3_ on Rh(111) is 40 kJ mol^–1^ more stable than the staggered confirmation. Agostic bonds have been observed between CH_3_ and Rh(111), Rh(211) and ad-row surfaces, except for the case of ad-atoms (Fig. 11). In the latter case, the CH_3_ binds to the ad-atom with C–Rh bond tilted to surface normal. The closest H–Rh interaction is 250 pm, indicating much weaker agostic bond interaction. Although the binding of CH_3_ is not the strongest, the ad-atom still can well stabilize the transition state. The D1 dissociation barriers catalyzed by ad-atoms are at least 20 kJ mol^–1^ lower than the corresponding barriers on Rh(111) and Cu(111) surfaces. However, the D2 barriers become comparable or even higher on ad-atoms compared with the corresponding barriers on (111) surfaces. In the final state of D2, the CH_2_ group attaches to bridge sites between the ad-atom (CN = 3) and a surface atom underneath (CN = 10), with an average CN = 6.5. Accordingly, the calculated binding energy of CH_2_ on the ad-atom/surface bridge site is similar to the value on step edges (CN = 7). Moreover, the dissociated H atom is about 20 kJ mol^–1^ less stable on top of ad-atoms than adsorption on other cases. The combination of both effects shifts up the D2 barrier on ad-atom.

A third type of studies covers the case with AB type alloy, in most cases with A : B = 1 : 1. Qi *et al.* ^56^ selected two metals, Pt and Ir, which are both active for CH_4_ dissociation. Instead of expected in between catalytic activity of alloy, the initial dehydrogenation activity is enhanced on PtIr(111), with a lower D1 barrier, 53 kJ mol^–1^, than barriers on Pt(111) (*E*_a_ = 82 kJ mol^–1^) and Ir(111) (*E*_a_ = 90 kJ mol^–1^). Similar lowest barrier on alloy surfaces is also observed for the D2 step. However for D3 and D4, the calculated barriers on PtIr(111) are quite similar to values on Ir(111), and lower than the ones on Pt(111). The enhanced activity on alloys does not seem to be unique for PtIr(111). Similar lower barriers on alloy surfaces were also observed on PdNi(111)^99^ and NiCu(111),^100^ while in the case of NiCo(111),^101^ the barriers are similar to those on Ni(111).

#### 3.2.2 Propane dehydrogenation on alloys

**Fig. 12:**
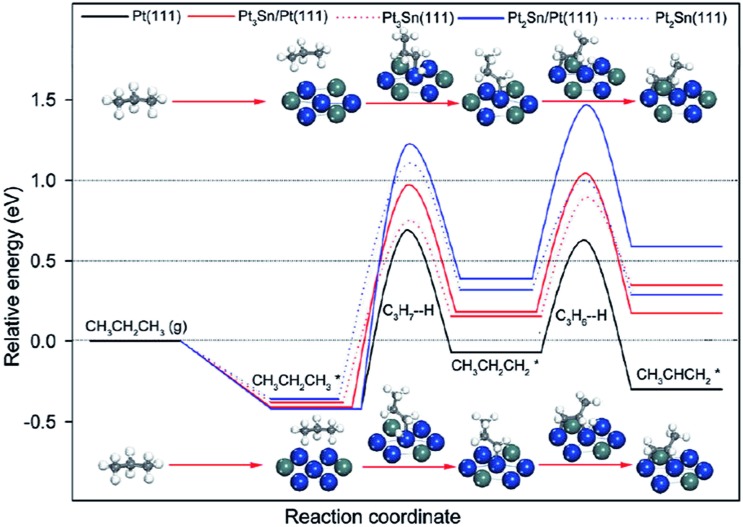
Energy profiles for propane dehydrogenation to propylene on Pt and PtSn surfaces. Reprinted with permission from ref. 104. Copyright 2012 American Chemical Society.

Previous experimental studies have shown^102,103^ that the selectivity towards propylene during propane dehydrogenation on Pt particles can be increased by alloying late transition metals or main-group metals such as Cu and Sn. For example, Han *et al.* have shown that the selectivity towards propylene increases from <80% achieved on supported Pt catalyst to about 90% after Cu is added to Pt during the catalyst preparation. The Pt–Cu catalyst inhibits the adsorption of formed propylene, and thus suppresses the secondary cracking reactions from propylene towards lighter hydrocarbons. Thus, this reduced interaction between the product and the catalysts enhances the anti-coking ability of the catalyst.^102^ Similar increased selectivity towards the desired propylene as achieved on the Pt–Cu catalysts was also reported for the reaction on Pt–Sn/Al_2_O_3_.^103^ Indeed, DFT calculations^104–106^ have shown that the binding energy of propylene is weakened by at least 8 kJ mol^–1^ after alloying different amounts of Sn in Pt(111). Meanwhile, all the dehydrogenation barriers shift up (Fig. 12), including deep dehydrogenation from propylene. In combination with the above two factors, the selectivity towards propylene on PtSn alloy is expected to be higher than that on pure Pt, although the total activity might be lower in the case catalyzed by PtSn. Moreover, on stepped (211) surface, Sn atoms are more preferred at step edges in PtSn alloy,^105^ which significantly reduces the activity of deep dehydrogenation and cracking of propane on step edges. Similar as the discussion of alloy for methane dehydrogenation, the lower reactivity (*i.e.* higher dehydrogenation barrier) on PtSn alloy is due to the deeper d-band center compared to pure Pt.

### 3.3 Metal clusters/particles

In addition to the slab models, another widely used approach is the cluster model, in which the catalyst is described by a finite metal particle. The advantage of the cluster model is that various types of site, *e.g.* corner, edge as well as terrace, can be described simultaneously in one model. In addition, it is easy to set up a supported particle calculation. The size of metal particle is normally limited to hundreds of atoms, whose diameter is in range of several nanometers.

**Fig. 13:**
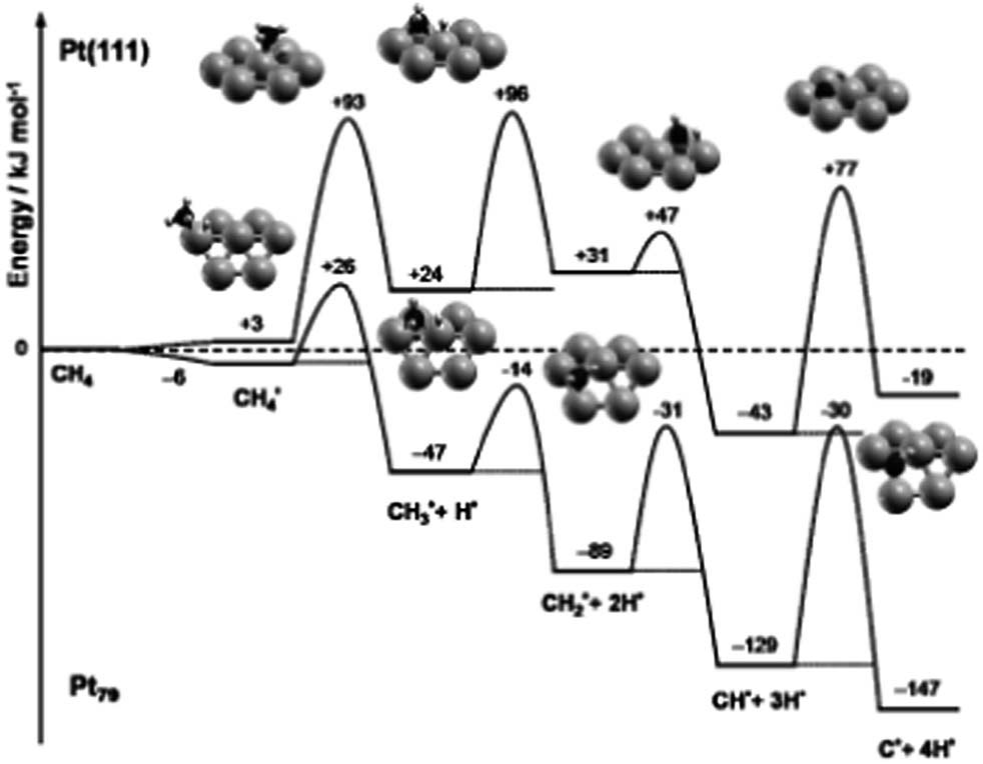
ZPE-corrected reaction energy profile for the complete dehydrogenation of methane on a Pt(111) surface and on a Pt_79_ nanoparticle. All energies, in kJ mol^–1^, refer to methane in the gas phase and the clean substrate. Reprinted with permission from ref. 59. Copyright 2010 Wiley-VCH Verlag GmbH.

In case of finite particles, the binding strength is also correlated to the size of the cluster besides the influence caused by the difference on coordination number. For example, a linear relationship between the size of Pd particles and the corresponding adsorption energy of CO has been established by Yudanov *et al.* ^107^ The particle size affected binding energy is linked to the strain effects.^30^ Shorter Pd–Pd distance was observed in smaller Pd particle/clusters, which results in stronger binding of intermediates and transition states on small metal particles than on large ones and on edge sites of (211) surfaces. Viñes *et al.* ^59^ has shown that the complete dehydrogenation of CH_4_ to form C atom and 4H atoms is more exothermic by 128 kJ mol^–1^ on Pt_79_ than on Pt(111) (Fig. 13). Stronger binding of dehydrogenated intermediate on Pt_79_ significantly decreases the first dehydrogenation barrier of CH_4_, only 32 kJ mol^–1^ on Pt_79_, while this barrier is 90 kJ mol^–1^ on Pt(111). Correspondingly, CH_3_ was observed on small Pt particles in a direct dissociation process to undergo spontaneous, thermally induced dehydrogenation, even at surface temperatures as low as 100 K.^59^

Enhanced dehydrogenation by small nanoparticles is not unique for methane. Vajda *et al.* ^108^ reported that size-preselected Pt_8–10_ clusters are 40–100 times more active for oxidative dehydrogenation of propane than previous studied platinum and vanadia catalysts. Calculations with a tetrahedral Pt_4_ cluster indicate the barrier for the first dehydrogenation of propane decreased to 41 kJ mol^–1^. Furthermore, further C–C bond breaking as well as dehydrogenation of the CH_3_ group from propylene have much higher barriers (*e.g.*, over 100 kJ mol^–1^) than the first two dehydrogenation steps to form propylene, which is consistent with the experimentally observed high selectivity of propylene.^108^ The high barrier for the C–C bond breaking can be explained by the sp^3^ directionality of the orbitals on C compared with the spherical nature of the s orbital on hydrogen, which results in poorer overlap between adsorbate and the reaction site orbitals in the transition state for breaking of the C–C bond.^108^

## 4 Other catalysts

### 4.1 Oxides

**Fig. 14:**
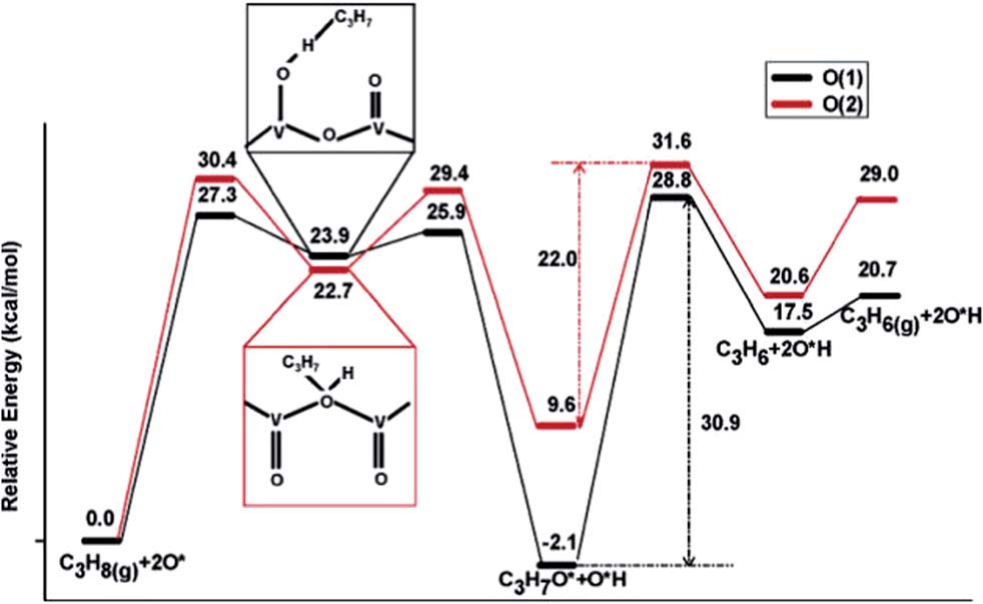
Lowest-energy pathways of propane ODH process on V_2_O_5_(001) occurring on O(1) (single coordinated O) and O(2) (di-coordinated O), respectively. Reprinted with permission from ref. 110. Copyright 2006 American Chemical Society.

Supported vanadium oxides are one of the best catalysts for oxidative dehydrogenation (ODH) of propane.^109^ It is generally accepted that this catalytic process proceeds *via* a two-step mechanism:^110^ (1) reduction of the oxide surface by dehydrogenation of hydrocarbon and (2) re-oxidation of the surface by gas-phase O_2_. At high vanadium loadings, vanadium oxide exists in form of V_2_O_5_, whose most stable surface is the oxygen terminated basal V_2_O_5_(001). There are three different types of surface O atoms. Depending on the type of the O center, the corresponding coordination number can be 1, 2 or 3. Previous experimental studies have suggested both the single coordinated^111^ and the di-coordinated^112^ surface O atoms to be the active site of ODH of propane. These suggestions are in line with DFT calculations by Fu *et al.*,^110^ which clearly showed that for propane activation on V_2_O_5_(001), the activation of a C–H bond by tri-coordinated O atoms is least likely, as yielded propyl upon dehydrogenation of propane is least stable at the tri-coordinated O sites on V_2_O_5_(001). On the other two O active sites, similar dehydrogenation barriers for the initial step were obtained, with slight preference for the single coordinated O (Fig. 14). However, the di-coordinated O has a higher activity for further decomposition of surface propoxide, *i.e.* the propyl bound to O site, into propylene. Nevertheless, one should be aware that a good catalyst for propane ODH should not only feature high activity for the dehydrogenation step but also yield a high selectivity towards desired product, *i.e.* propylene, over deep dehydrogenated ones. Unfortunately, these two properties of a catalyst are somewhat oppositional: a too high catalytic activity often comes with a decreased selectivity towards partially dehydrogenated propylene.^110^ Dai *et al.* ^113^ focused on ODH of ethane on vanadium oxide, whose rate was much lower than ODH of propane.^114^ DFT studies identified the ODH mechanisms from both ethane and propane are similar to each other: the first C–H dissociation step being rate-limiting in both cases. However, in the case of ethane, the undesired acetaldehyde can be formed on the single coordinated O site with a barrier that is slightly lower than the barrier associated with ethylene formation. This undesired acetaldehyde is a stable species on the surface. However, it was suggested, although without having explicitly calculated the corresponding elementary steps, that acetaldehyde can be further oxidized to CO or CO_2_ under the typical ODH reaction conditions. The presence of this easily accessible side reaction significantly lower the efficiency of ethane ODH, which was suggested to be the reason for the low ODH activity of ethane on V_2_O_5_.

**Fig. 15:**
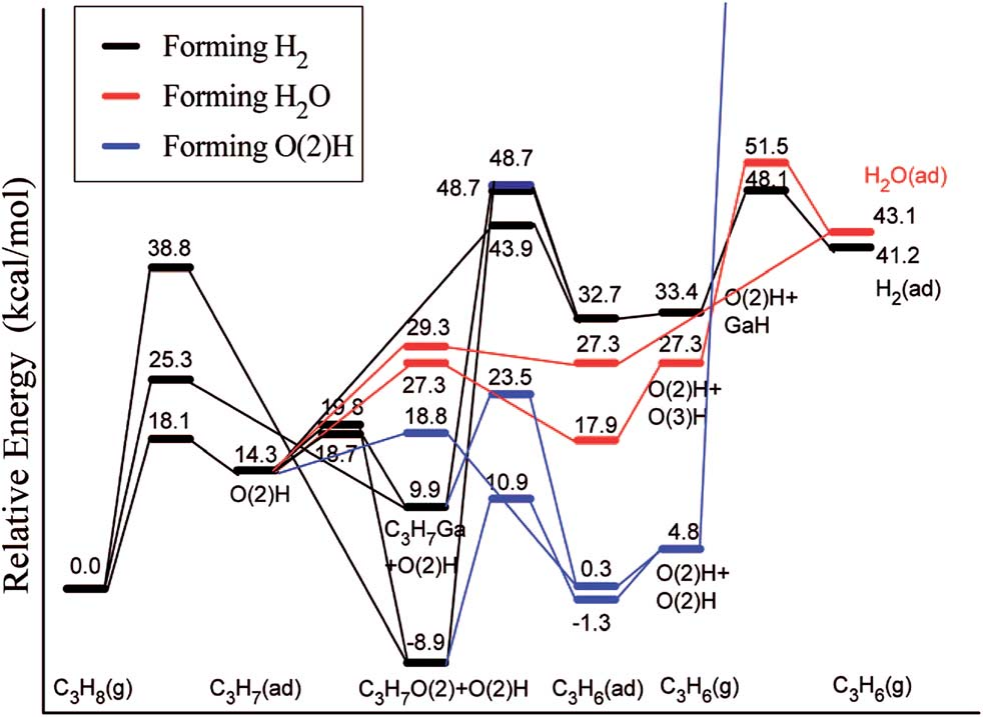
Lowest-energy pathways of propane ODH process on β-Ga_2_O_3_(100). Reprinted with permission from ref. 116. Copyright 2008 American Chemical Society.

Ga_2_O_3_ is another highly active and selective catalyst for the production of alkenes *via* alkane dehydrogenation reactions. However, such catalysts deactivate quickly, which was suggested to be a consequence of poisoning by carbon deposition formed in light alkane dehydrogenation reactions.^115^ Liu *et al.* ^116^ employed a slab model for β-Ga_2_O_3_ and considered two competitive mechanisms for dehydrogenation of propane, a direct dehydrogenation and oxidative dehydrogenation. The study reveals that the direct dehydrogenation mechanism is preferred over the oxidative dehydrogenation. However, the latter mechanism could not be completely ruled out for reactions in the presence of mild oxidants such as CO_2_. The most active site for the first dehydrogenation step is a bridge-bound surface O atom. Once the H is abstracted from hydrocarbon, rather stable surface hydroxyl groups are formed (Fig. 15). The direct removal of the H, either as H_2_ or H_2_O, to regenerate the O site of the catalyst is difficult and will eventually decrease the activity of the catalyst. Upon blocking of the O site by the H atom, Ga centers can also catalyze the further dehydrogenation of the intermediate propyl. This leads to the formation of a hydrogenated Ga center “GaH” and facilitates the removal of surface H at the neighboring O sites in the form of H_2_ or H_2_O.

We should note here that there is another important type of dehydrogenation catalyst which is based on chromium oxide. As early as the 1940s, UOP had already achieved the dehydrogenation of butane to produce butylene with chromia supported on alumina at industrial scale.^1^ Numerous experimental attempts have been performed in order to identify the active site and reaction mechanism for light alkane dehydrogenation reactions on chromium oxide, which was recently reviewed in ref. 1, and will not be repeated here. However, due to the complexity of the chromium oxide, including the different oxidation states, the crystal structure and the surface facets, theoretical studies addressing chromium oxide catalyzed dehydrogenation processes are challenging and thus rare.

### 4.2 Zeolites

Zeolites have been widely used as catalysts and/or supports for hydrocarbon cracking, alkylation, aromatization and isomerization reactions.^117^ Due to the nature of their 3-D structure, theoretical studies of this system either employ a finite cluster model describing the immediate surroundings of the active site,^118^ or use a 3-D periodic model to describe the complete structure of the zeolite system.^119^ At least two different mechanisms have been proposed by previous theoretical studies on zeolite catalyzed alkane dehydrogenation reactions: one proceeding *via* an alkyl intermediate and an alternative mechanism *via* a carbenium ion.^118^

**Fig. 16:**
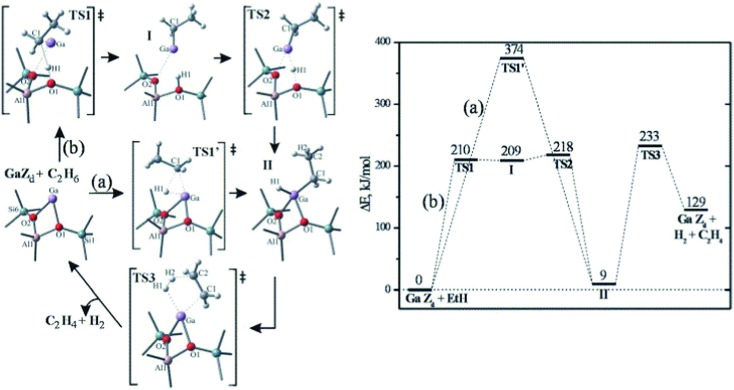
Homolytic (a) and heterolytic (b) “alkyl” pathways of ethane dehydrogenation over Ga in ZSM5. Reprinted with permission from ref. 118. Copyright 2006 Elsevier.

The dehydrogenation of alkanes in zeolites can be promoted by extra-framework metal atoms, such as Zn, In, Ga and Cu, which create new Lewis-acid sites in zeolites.^117^ However, the nature and role of the extra-framework metals are still under debate. Previous studies have suggested that metal oxide or metal hydride clusters are the active sites for dehydrogenation reactions. A series of studies on the dehydrogenation of light alkanes catalyzed by Ga exchanged ZSM-5 have been made by Pidko *et al.* ^118,120^ using a cluster approach. These studies considered four different possible species, Ga^+^, GaH_2_^+^, GaH^2+^ and GaO^+^, to be the active centers. Among the first three active sites, Ga^+^ is the most stable form of extra-framework Ga in ZSM-5. However, because the filled d and s orbitals of Ga^+^ are energetically low lying under the Fermi level, the Ga^+^ center is unable to donate or accept electrons, resulting in a high barrier (*E*_a_ = 374 kJ mol^–1^) for direct oxidative addition of ethane (Fig. 16).^118^ A two-step heterolytic splitting of the C–H bond is much more favorable with the highest barrier of 233 kJ mol^–1^ (Fig. 16), due to the polarization induced by the interaction of the hydrocarbon with the Ga···O Lewis acid–base pair. Hydrogenated gallium species are less active than Ga^+^ as reflected by the higher activation energies for ethane dehydrogenation. At variance, the oxidized gallium GaO^+^ shows the lowest initial dehydrogenation barrier of ethane (*E*_a_ ≈ 100 kJ mol^–1^) and yields very stable intermediates, [C_2_H_5_–Ga–OH]^+^ or [C_2_H_5_O–Ga–H]^+^. However, the desorption of C_2_H_4_ from both intermediates leads to formation of [H–Ga–OH]^+^, from which the regeneration of active site *via* H_2_ desorption is strongly disfavored both thermodynamically and kinetically.

For the Zn/ZSM-5 system, Pidko *et al.* ^121^ further considered a model with Zn at a distant location from the [AlO_2_]^–^ framework units. The initial dehydrogenation barrier of ethane on such a Zn site is lower than the barrier at Zn of a conventional ion-exchange site. This is related to the stronger Lewis acidity in the former case caused by the indirect charge-compensation.^121^ Once the [Zn–C_2_H_5_]^+^ is formed upon the dehydrogenation of ethane, the barrier of the following one-step elimination of H_2_ and C_2_H_4_ strongly depends on the relative position of [Zn–C_2_H_5_]^+^ and H^+^ species. Thus, the presence of acidic protons in the catalyst can promote the regeneration of active sites.^121^ Lower barriers for the initial dehydrogenation steps have been determined for the reaction at binuclear ZnOZn sites, which can be rationalized by the high Lewis basicity of the extra-lattice oxygen and strong steric stain of the active site. Both effects also lead to strong stabilization of the [Zn–C_2_H_5_···HO–Zn]^2+^ intermediate, resulting in a high activation barrier of 190 kJ mol^–1^ for the elimination of ethylene.

**Fig. 17:**
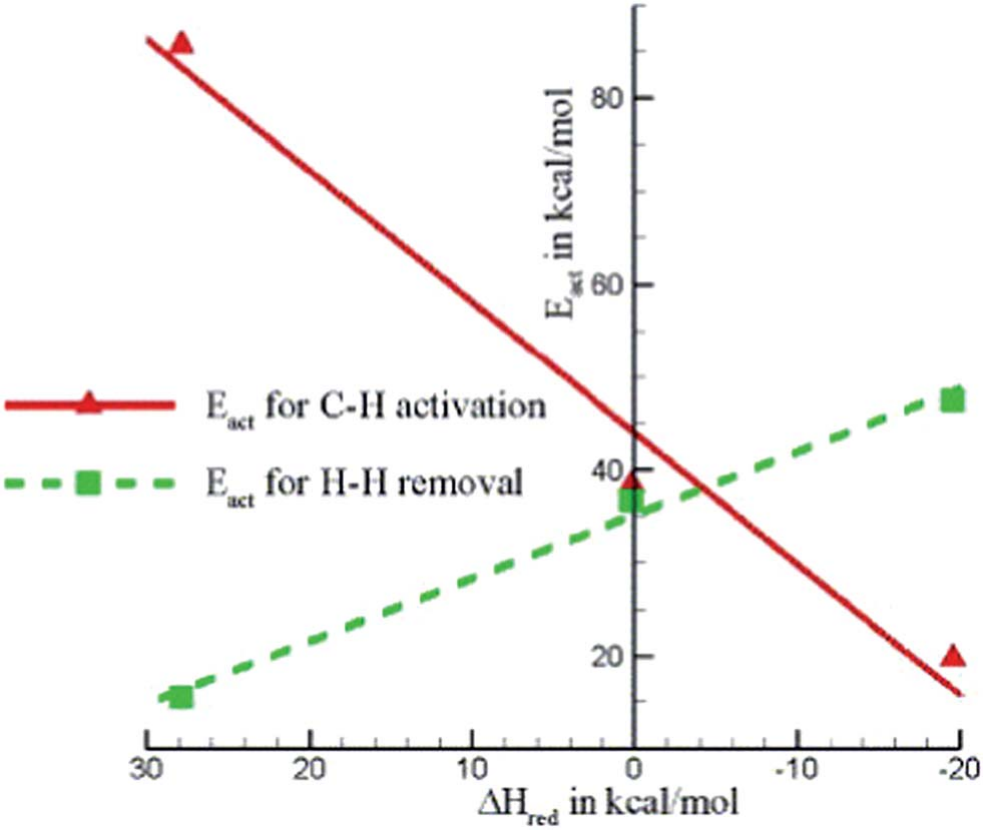
Important activation energies plotted against the heat of reaction for dissociative adsorption of H_2_ (*i.e.* reduction of the Z^2–^[GaH]^2+^species). Reprinted with permission from ref. 122. Copyright 2005 Elsevier.

The stability of metal oxides or hydride clusters is also influenced by the structure of the zeolite framework. Joshi and Thomson^122,123^ showed that [GaH]^2+^ is more stable in a six-membered ring structure than in an eight-membered ring.^122^ In the case of a more stable site, the interaction between reaction intermediates and active site is expected to be weaker. The poorly stabilized transition states result in a relatively high barrier for C–H activation as well as decreasing the catalytic activity of the system. On the other hand, the weak binding of the intermediate on such sites favors the desorption of H_2_. Considering these two opposite effects, calculations suggested that the optimal Al–Al distance to be 453 pm corresponding to a minimum overall barrier, and a simple ‘structure-to-activity’ correlation based on the Sabatier principle (Fig. 17) was proposed.^122^ Further thermochemical analysis indicates that pair-Al sites with larger Al–Al distances become more prevalent, which can be used as a guideline of optimal Si/Al ratio for a given Ga loading.^123^

Besides the position of Al atoms, the topology of zeolite framework also affects the dehydrogenation activity. Wannakao *et al.* ^124^ calculated the methane activation in Au-substituted FAU, FER, ZSM-5 and MCM-22. In FAU, Au binds to three O atoms, while it is bi-coordinated in the other three zeolites. The higher coordination number results in weaker binding of intermediates and transition states, leading to lower activity in FAU than in ZSM-5. Apart from the effect of the coordination of the extra-framework Au center, the pore size is also considered to have a slight effect on the catalytic activity, *i.e.*, lower activation barriers for reactions in zeolites with larger pore size.^124^

### 4.3 Single atom catalysts

Earlier calculations suggested initial dehydrogenation of alkanes in general requires a smaller ensemble of active metal centers, *i.e.* on top of a single atom, than deep dehydrogenations as well as C–C bond breaking, which normally require bridge or 3-fold hollow sites.^92^ Thus, the diluted concentration of surface active metal atoms suppresses deep dehydrogenation as well as coke formation, and in turn increases selectivity towards the desired alkenes. In the extreme case, dehydrogenation reactions may be catalyzed by an active site that composes of only one active metal atom on a support, which maximize the efficiency of metal utilization.^125^

**Fig. 18:**
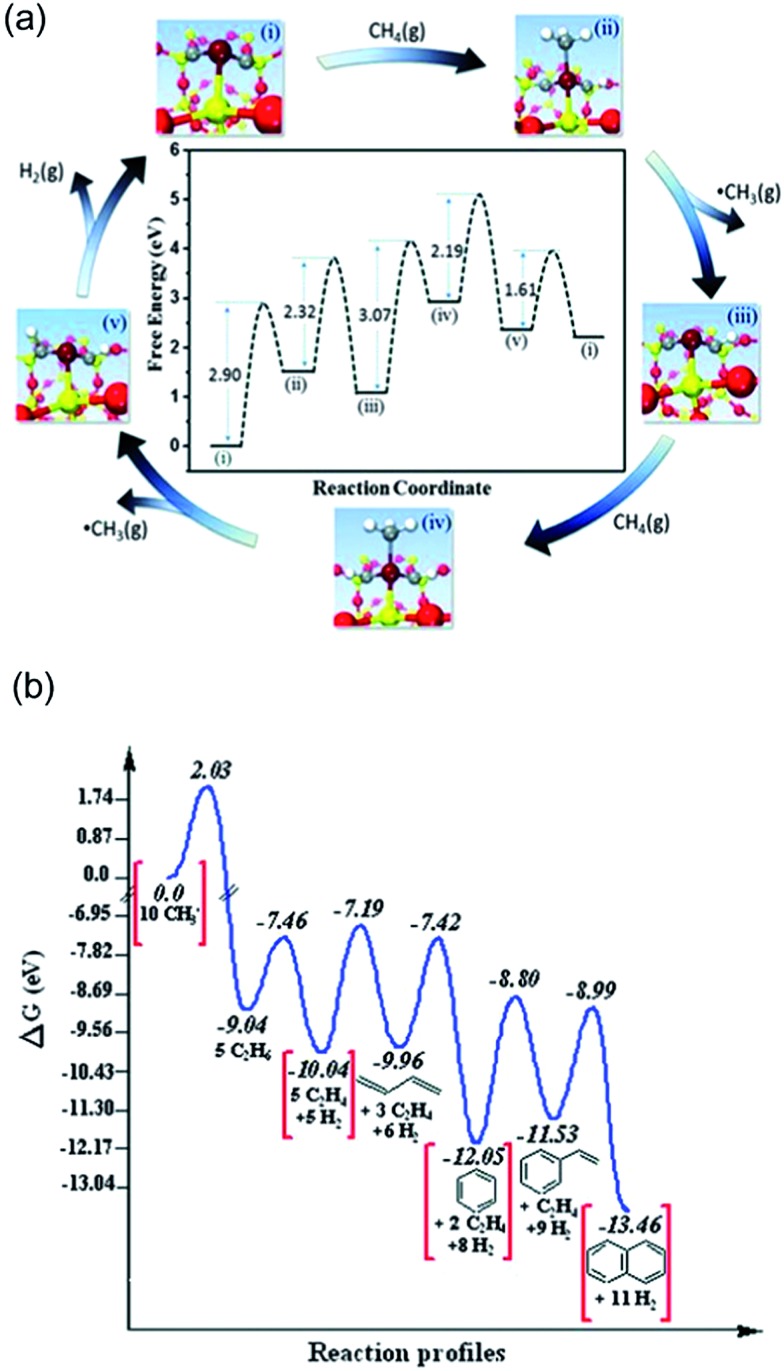
(a) DFT calculations on catalytic generation of methyl radicals at 1223 K. (b) DFT simulated reaction profile of methyl radicals in the gas phase at 1225 K; Δ*G*, Gibbs free energy. Reprinted with permission from ref. 126. Copyright 2014 American Association for the Advancement of Science.

Guo *et al.* ^126^ reported a direct, nonoxidative process for the conversion of methane to ethylene, aromatics and hydrogen that is catalyzed by a single Fe atom embedded in a silica matrix (Fig. 18a). Compared to other forms of the Fe catalyst, *e.g.* Fe supported on oxides or substituted in zeolites, the coke formation is negligible here. Based on DFT-calculated energetics, the active sites was suggested to be one Fe atom coordinated by one Si from silica supports and two C atoms originally derived from complete dehydrogenation of methane.^126^ It further indicates that the Fe center can active CH_4_ to form CH_3_, which desorbs as a radical into the gas phase at temperatures as high as 1200 K. A series of gas-phase radical reactions generate the final products, including ethylene and aromatics. Two CH_3_ radicals further combine to form ethane in a strong exothermic process, and ethane undergoes dehydrogenation to form ethylene with a barrier of 152 kJ mol^–1^. The aromatics can be formed by transformation *via* cyclization of C_2_H_3_ radicals generated from ethylene with high barriers, *e.g.* benzene (*E*_a_ = 275 kJ mol^–1^) and naphthalene (*E*_a_ = 314 kJ mol^–1^). Despite the aromatics being thermodynamically more stable than ethylene (Fig. 18b), the process can still be tuned to feature ethylene as major product by increasing the flow rate of the feedstock which reduces the secondary conversion from ethylene to aromatics.

**Fig. 19:**
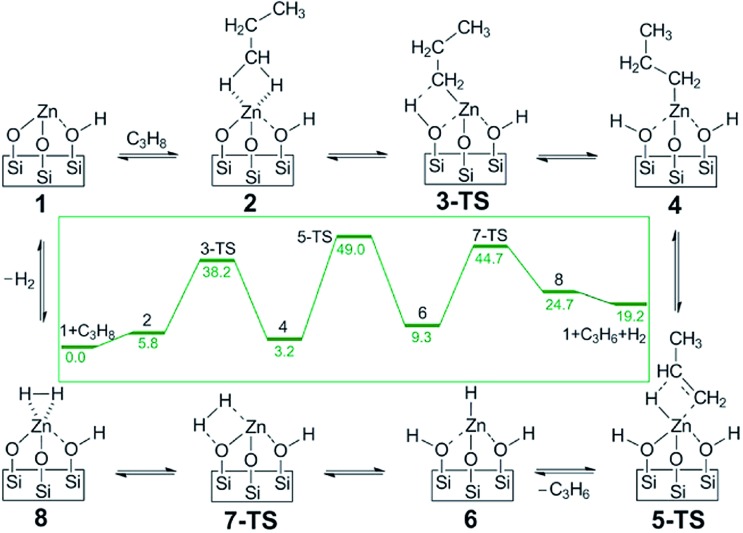
DFT calculated proposed catalytic reaction pathway for olefin hydrogenation and alkane dehydrogenation on single-site, Zn Lewis acid catalyst. The reaction free energies (kcal mol^–1^) are shown in the inset. Reprinted with permission from ref. 127. Copyright 2014 American Chemical Society.

Another example for a single atom catalyst is the Zn^2+^/SiO_2_ system used for selective dehydrogenation of propane to propene.^127^ In this catalyst, the Zn^2+^ center is coordinated with three O centers of the SiO_2_ surface. DFT-calculated barriers show the rate-limiting step is the second dehydrogenation with simultaneously desorption of propylene, with a barrier of 192 kJ mol^–1^ (Fig. 19). The C–C bond breaking step is at least 42 kJ mol^–1^ higher than dehydrogenation reactions, consistent with the high selectivity to propylene.

## 5 The origin of coke formation

**Fig. 20:**
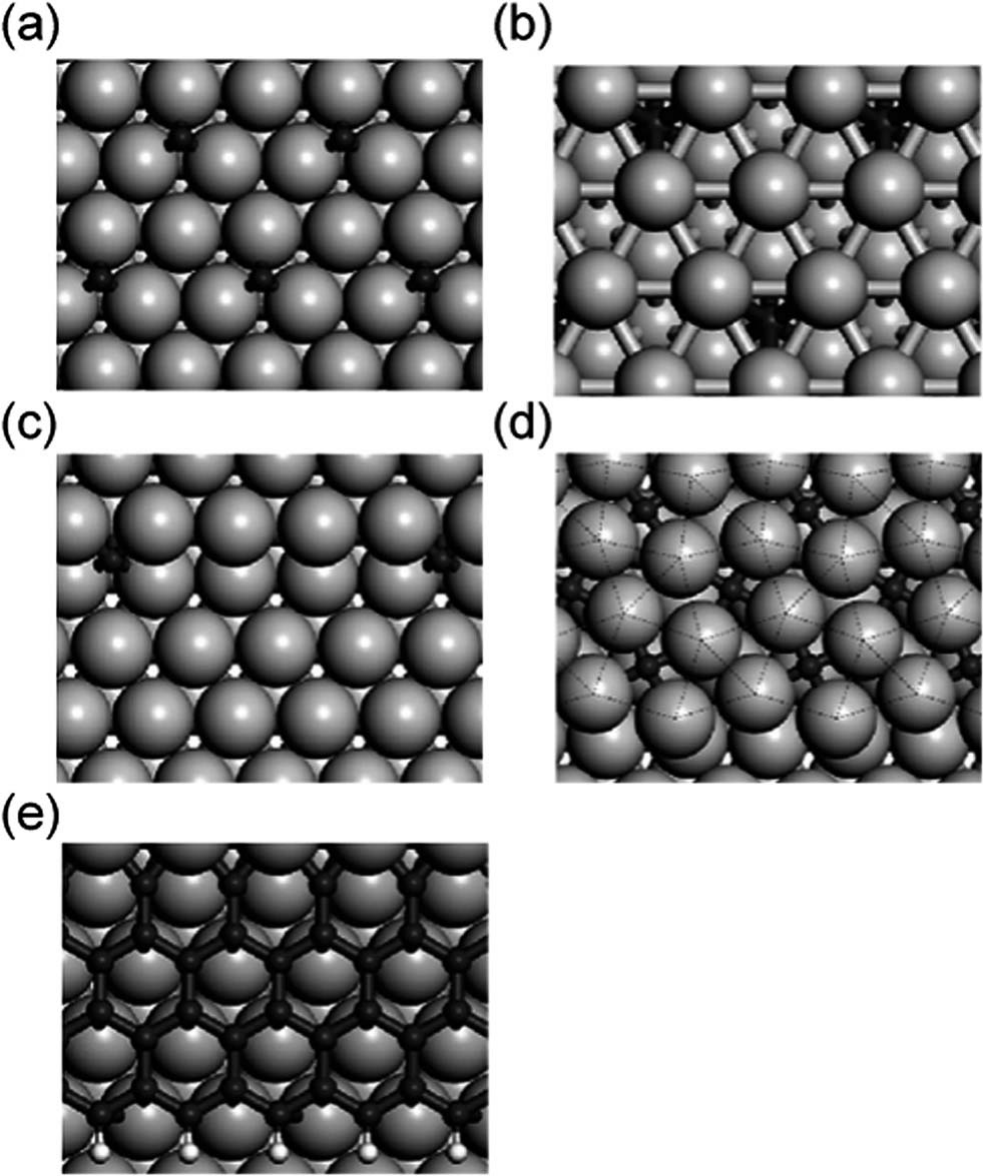
Different types of adsorbed C atoms on Co surfaces: (a) on-surface; (b) sub-surface; (c) step; (d) P4g clock; (e) graphene. Adapted with permission from ref. 140. Copyright 2010 American Chemical Society.

The following section deals with the side reaction of the dehydrogenation process leading to the formation of coke. Coke may, for instance, form if carbon centers are completely dehydrogenated but not removed from the catalyst surface. The unwanted formation of carbonaceous depositions not only decreases the selectivity towards the desired products, but also leads to the modification of a catalyst and the reduction of the catalytic activity up to complete deactivation. When discussing “coking”, one should be aware that the carbonaceous species can be present in different forms, which are of varying stability, *e.g.* as on-surface C atoms, as sub-surface C atoms, forming a carbidic phase, but also as graphene islands covering the catalyst surface (Fig. 20).^128^ Although this review intends to address the dehydrogenation of light hydrocarbons, this section will be kept more general and discuss the coking process in catalytic reactions that involve general organic species (*e.g.* steam reforming, Fischer–Tropsch synthesis, *etc.*). We will see in this section that the crucial processes for coking (or for the prevention thereof), are the reactions of the isolated C centers, which may arise from the dehydrogenation (and C–C scission) of hydrocarbons but also from CO activation as occurring in Fischer–Tropsch synthesis. As the origin of the C centers is not of primary importance for the discussion of the coking process, it seems reasonable to extend our focus on the coking process in systems that are beyond the typical catalysts used for alkane activation. In the following section we will begin the discussion exemplarily on the relatively well studied formation of coke on Ni surface, which is the most widely used reforming catalysts, and show the strategies proposed to enhance the robustness of Ni against coking.

### 5.1 Coking on Ni

Although there are other metallic reforming catalysts such as Ru and Rh, which are less strongly prone to coking than Ni,^129^ Ni is one of the most common reforming catalysts, which is partly due to its significantly cheaper price. In order to overcome the coking problem of Ni catalysts, numerous theoretical studies have been conducted to understand the coke formation on nickel and to come up with potential strategies to avoid the unwanted coking process.

#### 5.1.1 Coke formation mechanism

**Fig. 21:**
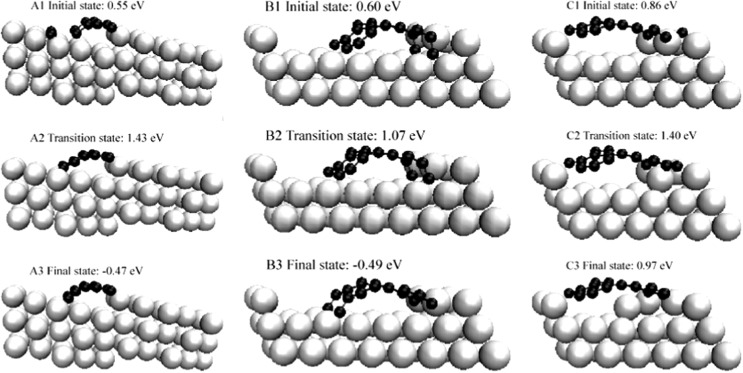
Initial transition and final states of three pathways on growth of graphic structure. Reprinted with permission from ref. 130. Copyright 2006 American Physical Society.

The formation of graphene-island type coke on Ni is analogue to the growth of carbon nanotubes.^130^ This reaction has been described as a process consisting of multiple steps^128,130^ starting with the decomposition of carbon containing gas-phase species yielding C centers on the catalyst surface. These carbon centers can dissolve into the sub-surface layers of Ni and diffuse to those facets of the catalysts that are suitable for the graphene growth. In addition to the sub-surface diffusion of C, a transportation mechanism *via* an on-surface diffusion has also been discussed.^130^ Once the C centers agglomerate at suitable facet sites, graphene islands can be formed and eventually deactivate the Ni catalyst by encapsulating the metal surface. It is known that the formation of the graphene islands on Ni is a structural dependent process, which requires step sites on the Ni surface.^66^ A detailed investigations by Abild-Pedersen *et al.* ^130^ addressing the formation of carbon nanotubes on Ni has revealed that the step sites on Ni surfaces are the thermodynamically most preferred adsorption sites for C atoms and act as the growth centers for the graphene islands. Three different mechanisms, which all feature similar total barriers around 140 kJ mol^–1^, have been identified for the growth of graphene type structures on Ni step sites: (A) the addition of C centers to the graphene island formed on the (111) facet of the Ni, (B) the incorporation of C atoms at the step edge into the growing graphene island, and (C) the exchange of C atoms with step-edge Ni atoms that are attached to the edge of the graphene islands (Fig. 21).^130^ Note that the formation of the graphene island is essentially independent from the dehydrogenation steps, whereas the transportation of the C atoms and the growth of the graphene islands are the crucial molecular processes.^130^ In other words, the coking process discussed here also applies for other processes in which surface C centers may form from reactions other than the dehydrogenation steps, *e.g.* decomposition of CO or alcohols.

It has been demonstrated for graphene islands on the Ni(111) surface, unsaturated C atoms at the edge of a graphene island are less stable than isolated C atoms on the metal surface.^66,128^ At variance the atoms “inside” the graphene islands coordinated by three other C atoms are more stable than the isolated C.^66,128^ Consequently, graphene islands are only stable, if they reach a critical size. Depending on the model and the assumptions, this critical size has been estimated to be between 10 to 80 C atoms.^66,128^ Thus, it has been concluded that the formation of graphene islands is a process with a high reaction-order whose formation is most favourable at high C coverage rates.^128^

A second form of coke on Ni is the carbidic layer. In such structures, C atoms occupy sub-surface sides. In contrast to the formation of graphene-islands, the diffusion of C atoms to sub-surface sides forming a carbidic layer is discussed to be a first-order process with an associated barrier estimated to be around 70 kJ mol^–1^.^128^ In other words a carbidic layer can also be formed at low C coverage on the surface. Also this type of coke has shown to reduce the catalytic activity of Ni for methane reforming: the barrier of methane activation on a Ni(111) surface with sub-surface C is reported to be 143 kJ mol^–1^, significantly higher than the value of 91 kJ mol^–1^ calculated for the analogue process on a pure close-packed Ni surface.^131^ A similar trend is also found, when comparing the methane activation on pure Ni surfaces with the reaction on surfaces of the Ni_3_C system.^132^

#### 5.1.2 Strategies against coking

In order to explore computationally the robustness of a catalytic system against coking, different parameters have been proposed as a measure for the susceptibility of a system to coking. One of the earlier studies have proposed to consider the C adsorption energy,^134^ while a more recent study advocated the consideration the rate ratio for the oxidation of surface CH and C groups (*r*_CH_/*r*_C_) as well as the rate for the cleavage of carbon monoxide.^132^ The idea behind the investigation of the *r*_CH_/*r*_C_ can be rationalized as follows: a low *r*_CH_/*r*_C_ ratio means that the oxidation of the carbonaceous species mainly proceeds *via* the intermediate formation of isolated C centers, which is also the main precursor for the formation of coke.^132^ The activation barriers for the oxidation of CH and C implies that the close-packed Ni(111) surface is more robust against the accumulation of isolated C atoms and thus also against coking than the stepped Ni(211) surface or the stepped (001) and flat (111) surfaces of the carbidic Ni_3_C system. It is interesting to note here that the flat Ni(111) seems generally less prone for the accumulation of C centers, irrespective of its origin. It has been shown that during the Ni-catalyzed water–gas shift reaction, less coke is expected to form at terrace sites of the catalyst compared to the situation at steps, as the flat surface is less active for the formation of C centers *via* CO scission.^133^

Different approaches have been proposed to encounter the coking problem. It could be shown that the adsorption of C atoms on the closed packed surface of a NiAu surface alloy is thermodynamically less favoured, by 23 kJ mol^–1^ or more, than the analogous process on a pure Ni(111) surface.^134^ Based on this finding, it has been proposed to alloy Ni with Au.^134^ A cheaper alternative is to alloy Ni with Sn which hinders the C–C bond formation and enhances the C–O bond formation leading to the removal of surface C centers as carbon monoxide.^135,136^ It has been discussed that the Sn atoms separate the Ni centers on the catalyst surface, which tend to bind C atoms, from each other. With the Ni centers being separated, the C atoms on the surface are separated as well, which hinders the formation of coke.^135,136^ These theoretical results are consistent with experiments which show that pure Ni catalysts deactivate significantly faster than NiSn alloy based catalysts.^135^

Since the step sites play an essential role in the growth of the graphene islands, as mentioned above, additives such as potassium,^66^ sulfur,^66^ gold^66^ or boron^131^ have been proposed.^66^ Computational calculations have shown that these elements tend to occupy the step sites and thus avoid the formation of graphene islands.^66,131^

A similar strategy, by blocking the crucial sites, has been proposed to prevent the formation of a carbidic phase. Calculations have shown that boron prefers the same sub-surface sites on Ni as C.^137^ By occupying these sites with B, the diffusion of C to these sub-surface sites and the formation of the thermodynamically more stable carbide phase can be prevented.^128^ Apart from blocking the sub-surface sites, sub-surface boron in Ni(111) also has been shown to have an destabilizing effect on on-surface C centers,^138^ which may help to prevent the accumulation of C atoms on the surface.

### 5.2 Coking on other metals

Similar to Ni, experimental and DFT studies on Co have shown that coke can be present as graphene islands as well as in the form of a surface carbide.^139^ The comparison of adsorption properties of boron and carbon on Co surfaces reveals that both elements behave similarly, *i.e.* both elements occupy the same adsorption sites. This indicates that the presence of B may prevent the deposition of carbonaceous species on Co,^140^ comparable to the case for Ni described above. This hypothesis, based on theoretical consideration, could be confirmed by experiments, which demonstrated that the promotion of a Co Fischer–Tropsch catalyst with boron can significantly reduce the deactivation rate, while leaving the selectivity and activity unaffected.^140^

It is interesting to note here that also the presence of on-surface carbonaceous species of the formula CH_*x*_ (*x* = 0–3) have been discussed to influence the activity of a catalyst. An example is the decomposition of methanol studied on Pd cluster models.^141^ It could be shown that CH_3_ and CH_2_ species formed from the C–O cleavage of methanol tend to move to the thermodynamically more stable near-edge sites of the cluster where they further dehydrogenate to CH or C.^141^ The calculated results are in line with experimental studies which indicate that the decomposition of methanol leads to the formation of carbonaceous species at the near-edge sites of the Pd catalyst.^142^ The experiments further demonstrates that the occupation of the near edge sites leads to a strong decrease of the C–O cleavage rate, while the complete dehydrogenation of methanol yielding CO is essentially unaffected.^142^ This finding shows that the formation of carbonaceous species is not generally negative. In the above example, the on-surface C centers selectively block the C–O cleavage reaction which is for instance unwanted in methanol reforming.

A study addressing reactions over Pt clusters supported on γ-alumina has compared the Gibbs free energies of reaction for alkane dehydrogenation and for the undesired coke formation and hydrogenolysis processes.^143^ By considering the Δ*G* values, the impact of the alkane pressure as well as of the H_2_ pressure could be investigated. It has been demonstrated, in accordance with experimental observations, that the performance of the dehydrogenation process is the best, if the ratio between the H_2_ pressure and the alkane pressure is between 1 and 10.^143^ At lower H_2_ : alkane ratios, the formation of coke precursors *via* highly dehydrogenated carbon species is promoted, whereas at larger H_2_ : alkane ratios, the dehydrogenation of the alkane is hindered. Despite the fact that this result appears rather intuitive, it should be noted that the underlying molecular processes are far more complex. An example is the destabilization of C_1_ C–C scission products: the formation of the C–C scission products induces a change in the geometry of the Pt cluster. The so-formed cluster morphology binds relatively little hydrogen atoms, which leads, under high H_2_ pressure, to a high and thermodynamically unfavoured value for the Gibbs free energy.^143^

Although we have mentioned in the beginning of this section, that Ru and Rh catalysts are more robust against coking than Ni, carbon deposits can also form on surfaces of such metals. It has for instance been reported, that Rh(111) surfaces can be used for the growth of graphene.^86^ Calculations on Ru have shown that C atoms on a Ru surface tend to occupy the step sites, which are about 100 kJ mol^–1^ more favoured than the terrace sites.^144^ Experiments have shown that this deactivates the highly structure sensitive CO scission,^144^ a process that plays a central role in the methanation process or the Fischer–Tropsch process.

## 6 Concluding remarks and perspectives

With state-of-art DFT calculations, more and more insights into the mechanistic aspect for heterogeneous catalytic C–H activation reactions have been gained in the last two decades. The most widely calculated model system, methane dehydrogenation on metal surfaces, has covered many important transition-metal catalyst as well as alloy systems. Although most of the calculations have been conducted on relatively simple slab models, these results still serve as important clues which successfully explain many experimental phenomena. Similar to many heterogeneous catalyst reactions, the dehydrogenation activity is observed to follow the d-band rule: a surface with higher d-band center tends to bind intermediates and transition states more strongly, in general resulting in a low dehydrogenation barrier. Although low dehydrogenation barrier indicates high dehydrogenation activity for a catalyst, too high dehydrogenation activity might result in uncontrolled deep dehydrogenation of hydrocarbons, which in the worst case results in undesired coke formation.

The ultimate goal for theoretical studies is to identify optimal catalysts for particular chemical processes. Recently developed BEP or other linear scaling relationships connect transition states, which are computationally expensive to be located, with simply calculated parameters or so called descriptors, such as reaction energies or binding energies. These linear relationships reduce the complexity of a catalytic system from a high dimensional system with rate constants of *N* elementary steps to a few, in many cases even with only one or two, dimensions of descriptors, which offers a powerful strategy to achieve fast screening of large numbers of new catalysts.

The continuing improvements of computer hardware, the development of new functionals, as well as the increasing sophistication of computational electronic structure software have insured the ability of a computational study to access more complicated catalytic systems beyond simple transition-metal surfaces. Although the general trend of catalytic activity is not as easy to be addressed for these as for metal systems, the case studies still shed light on dehydrogenation mechanisms in these systems, and more insights emerge which are normally difficult to be explored at molecular level even by the most advanced experimental equipment.

However, there are still limitations for current theoretical calculations to model more realistic catalytic systems. For example, there is still no perfect solutions to calculate entropies of soft modes, including frustrated translation and rotation, for an adsorbate, which prevents an accurate estimation of surface thermodynamics. The above mentioned linear scaling relationships introduce additional errors besides the errors by DFT calculations. In addition, most of the linear scaling relationships have been developed at a given coverage of a single adsorbate. However, under reaction conditions, the catalyst surface is a complicated system with different types of co-adsorbates. Although advanced kinetic modeling does explicitly include co-adsorbates, a fast and reliable way to generate binding energies as well as reaction barriers with different types of co-adsorbate is still under development.

In more complicated cases such as oxides, the reacting molecules can induce change of the structure of a catalyst, creating vacancies or oxidizing surface metal atoms, resulting in a large amount of different types of sites for elementary reactions, and dramatically increase the computational complexity. Moreover, many catalytic reactions are catalyzed at the interface of a bi-functional catalyst, which is also not easy to be well described by calculations. The lattice mismatch between the particle and support results in a shift of relative positions between sites on particle and surface, which results in different environments of given sites, leading to a large number of possibilities to be considered in a single study. Similar issues occur in the case of zeolites, where the possibility to distribute Al atoms in the framework increase rapidly with increase of Al/Si ratio. The complexity of the systems mentioned above seems to beyond the ability to completely scan the potential energy surfaces to a large extent with DFT calculations, which limits most of the current studies which focus to understand one aspect of the catalyst instead of the complete picture of dehydrogenation. There is still a long way to go for theoretical studies to generate a complete and accurate description of complex catalytic systems.

In addition to the development of computational approaches, the progress of advanced *in situ* characterizations has helped in the identification of active sites in complex catalytic systems and offers more guidelines for theoretical studies to build more reliable models. On the other hand, controllable synthesis *via* colloidal chemistry is also necessary which can maximize the content of the theoretically identified highly active and selective sites and structures in a real catalyst.
